# Nucleolar aggregation of key neuropathological proteins in the postmortem neurodegenerative brain

**DOI:** 10.1007/s00401-025-02968-2

**Published:** 2025-12-03

**Authors:** Guinevere F. Lourenco, Maria Elizabeth Torres-Pacheco, YuHong Fu, Hongyun Li, Heather McCann, Claire E. Shepherd, Jillian J. Kril, Glenda M. Halliday

**Affiliations:** 1 Neuroscience, School of Medical Sciences, Faculty of Medicine and Health, University of Sydney, Sydney, Australia; 2 Brain and Mind Centre, University of Sydney, 100 Mallett Street, Camperdown, NSW 2050 Australia; 3 Neuroscience Research Australia, Sydney, Australia; 4 UNSW Medicine and Health School of Biomedical Sciences, UNSW Sydney, Sydney, Australia

**Keywords:** Neurodegeneration, Nucleolus, Nucleolar aggregation, Nucleolar aggresomes, Nucleolar amyloid bodies, Nucleolar cavities, Nucleolar sequestration

## Abstract

Nucleolar disturbances have long been implicated in neurodegenerative diseases but, to date, aggregation and immobilization of proteins into nucleolar bodies have only been reported in vitro and in cell models, and only for amyloid β (Aβ). In model systems, these bodies have been shown to coordinate local nuclear protein synthesis with potential to seed diagnostic neuropathologies. Here we confirm the presence of nucleolar aggregates of amyloid nature in postmortem brain tissue from controls and patients with neurodegenerative pathologies and demonstrate the nucleolar sequestration of fibrillation-prone proteins associated with neurodegenerative diseases (Aβ, tau, α-synuclein, TDP-43, and FUS, but not prion or peptide repeats). We identified nucleolar bodies ranging from multiple small foci to a centralized, large amyloid aggresome, that appear to represent progressive stages of protein immobilization from liquid-like foci to the formation of nucleolar aggresomes. Neurons with nucleolar aggresomes were more vulnerable to neurodegeneration, decreasing in number with increasing duration of disease. Nucleolar aggresomes with phosphorylated tau correlated with increasing amounts of neuropathology, while phosphorylated TDP-43 in nucleolar aggresomes distinguished cases with limbic-predominant age-related TDP-43 encephalopathy. Nucleolar aggresomes containing α-synuclein occurred in a large proportion of aged controls with limited neuronal loss (potentially asserting neuroprotection). Other fibrillation-prone proteins were either absent (prion and peptide repeats) or found less commonly in nucleolar aggresomes (Aβ and FUS), and amyloidogenic nuclear proteins not screened in this study may also occur in nucleolar aggresomes. Our data do not support the concept that proteins in aggresomes seed diagnostic neuropathologies as there were no associations between their presence in nucleoli aggresomes and their cytoplasmic or extracellular accumulation. Assessment of neurons with and without phosphorylated tau or α-synuclein aggresomes showed that phosphorylated tau ameliorated the increased DNA levels found in AD. Collectively, our observations establish that nucleolar sequestration of amyloidogenic proteins is a common molecular mechanism in the brain, representing a novel contribution to the understanding of nucleolar protein aggregation in the context of neuroprotection and neurodegeneration during brain aging.

**Supplementary Information:**

The online version contains supplementary material available at 10.1007/s00401-025-02968-2.

## Introduction

The nucleolus, the most prominent nuclear substructure, is a multilayered biomolecular condensate built around nucleolar organizer regions—ribosomal DNA (rDNA)-containing regions located on the short arms of human chromosomes 13, 14, 15, 21, and 22 [91]. The nucleolus is composed of three distinct, phase-separated subcompartments arranged like nesting dolls: the fibrillar center (FC) at the inner core, the dense fibrillar component (DFC), and the granular component (GC). Ribosomal RNA (rRNA) synthesis by RNA polymerase I occurs at the FC–DFC interface, with nascent transcripts initially undergoing pre-rRNA processing in the DFC while further maturation steps, including the assembly of ribosomal ribonucleoprotein (rRNP) particles, occur in the GC [33]. However, only 30% of nucleolar proteins are involved in ribosome biogenesis, and it is now considered more critical in controlling protein trafficking during stress [33].

Prolonged stress and accumulation of neurodegeneration-related peptides, however, can lead to the formation of more stable aggregates either outside or inside the nucleolus. In fact, disruption of nucleolar phase transition in cell lines and animal models expressing dipeptide repeat proteins (DPR) produced by repeat-associated non-ATG (RAN) translation of an intronic hexanucleotide repeat expansion in the *c9orf72* gene—the most common genetic cause of familial amyotrophic lateral sclerosis (ALS) and frontotemporal lobar degeneration (FTLD) [20, 76]—causes DPR aggregation in the nucleoplasm, inhibits ribosome biogenesis, and results in cell death [28, 45, 52]. The recruitment and immobilization of pathological Aβ peptides into nucleolar amyloid bodies (A bodies) have been reported in vitro and in cell models [8, 9, 16, 97], but verification of Aβ nucleolar sequestration in postmortem human brain has not been demonstrated. As the nucleolus undergoes continuous protein exchange with nucleoplasm and even the cytoplasm under various cellular stressors, it can produce diverse transient physiological A bodies that coordinate local protein synthesis [11, 28, 29, 48, 63, 92, 97].

This study initially aimed to confirm the presence of nucleolar aggregates of amyloid nature in the postmortem brain from patients with Alzheimer’s disease (AD) and other neurodegenerative diseases. Since nucleolar immobilization relies on the fibrillation-prone properties of intrinsically disordered proteins, we then investigated if key neuropathological proteins (Aβ, tau, α-synuclein, TDP-43, FUS, prion protein, DPR, and polyglutamine tract) that form amyloid-like fibrillary structures [3, 72, 88] also undergo nucleolar sequestration. We also assessed the relationship between nucleolar sequestration of these proteins and the different cytoplasmic and extracellular diagnostic pathologies these proteins form. In particular, we investigated whether nucleolar aggregates occur first potentially seeding cytoplasmic and/or extracellular diagnostic neuropathologies, i.e., act as initiators of the characteristic lesions that define each disease where an upstream aggregate initiates downstream diagnostic pathological lesions, analogous to the regional prion-like propagation described for tau, α-synuclein, TDP-43, and Aβ [13]. We identified large, central nucleolar *cavities* containing amyloids (but not DNA or RNA) associate with neurodegeneration, and showed that only some neuronal aggregating proteins are consistently found in these structures (Aβ, phosphorylated tau, α-synuclein and phosphorylated TDP-43), that only certain case types concentrate nucleolar aggresomes with particular aggregation-prone proteins (α-synuclein in controls and phosphorylated TDP-43 in limbic-predominant age-related TDP-43 encephalopathy (LATE)), and that phosphorylated tau-containing aggresomes increase with increasing neuropathologies occurring in neurons with normalized levels of nuclear DNA. The deposition of cytoplasmic protein aggregates was independent of their nucleolar deposition in aggresomes. Together, these observations are consistent with phase separation of proteins in the nucleolus of neurons under different circumstances and to different degrees, with some protein aggregates associated with progressive neurodegenerative disease, while some appear involved in neuroprotection or repair.

## Materials and methods

### Demographics, clinical, and neuropathological information

All brains used in this study were collected by the Sydney Brain Bank following informed consent. Diagnostic slides were made available for confirmed cases with pathological AD with or without LATE (AD ± LATE, *N* = 35), AD with Lewy body disease [(AD + LBD), *N* = 9)], Lewy body disease (LBD, *N* = 29), FTLD with tau pathology (FTLD-tau, *N* = 20), FTLD with TDP-43 pathology (FTLD-TDP, *N* = 15), FTLD with FUS pathology (FTLD-FUS, *N* = 5), Huntington's disease (HD, *N* = 5), LATE (*N* = 8), and aged controls (*N* = 7). Diagnostic slides available for analysis were from the frontal, temporal, and anterior cingulate cortices and immunostained for Aβ, phosphorylated TDP-43, pan- and phosphorylated α-synuclein, phosphorylated tau, FUS, prion, poly(GA), and polyglutamine proteins with hematoxylin counterstaining (details below). Not all brain regions were available for all cases. Neuropathological staging was recorded for AD neuropathological change using the ABC scoring system [36]—the *A* score reflects amyloid β plaque deposition, ranging from A0 (no Aβ pathology) to A3 (widespread cortical deposition), the *B* score reflects the extent of neurofibrillary tangle pathology according to Braak staging, ranging from B0 (no tangles) to B3 (extensive neocortical involvement), and the *C* score reflects neuritic plaque density, ranging from C0 (none) through C1 (sparse) and C2 (moderate) to C3 (frequent)—for Lewy body stage using the Braak staging system [12]—0 (no pathology), 1–3 (brainstem pathology with minimal cell loss), 4 (brainstem pathology with cell loss), 5 (additional limbic pathology), and 6 (additional neocortical pathology)—and for TDP-43 stage using the LATE consensus staging system [71]—0 (no pathology), 1 (amygdala only pathology), 2 (additional hippocampal pathology), and 3 (additional frontal pathology). Full demographics, clinical, and neuropathological information is provided in supplementary Table 1.

For the analysis of the relationship between poly(GA) and the presence of nucleolar *cavities*, a separate cohort was made available (supplementary Table 2) which included tissue sections from the cerebellum of confirmed cases with pathological *c9orf72*-related FTLD-TDP (*N* = 17) and *c9orf72*-related ALS (*N* = 11).

### Immunohistochemistry

FFPE 10-μm sections from all available regions were immunostained with antibodies for nucleolar, nuclear and pathological proteins, as detailed in supplementary Table 3. Formic acid pretreatment was carried out for sections to be stained with anti-Aβ, anti-α-synuclein, and anti-phospho-α-synuclein antibodies. Tissue sections immunostained with anti-phospho-TDP-43, anti-α-synuclein, anti-phospho-α-synuclein, anti-FUS, anti-polyglutamine-expansion, and anti-prion protein antibodies also underwent heat-induced epitope retrieval pretreatment with a pH 6.0 citrate buffer. Immunohistochemistry was performed with a VENTANA BenchMark GX autostainer (Ventana Medical Systems, Tucson, AZ, USA) and visualized with either OptiView DAB IHC Detection Kit (Roche Diagnostics, Cat. No. 760-700) or with NovoLink Polymer Detection System (RE7270-CE, Leica Biosystems, Wetzlar, Germany) and counterstained with hematoxylin according to the manufacturers’ instructions. DPX mounting media (Sigma-Aldrich, Cat. No. 317616) was used to coverslip sections.

### Congo red staining

Formalin-fixed paraffin-embedded (FFPE) temporal cortex sections (10 μm) from aged controls (*N* = 5), LATE (*N* = 5), pure AD (*N* = 4), AD + LATE (*N* = 5), AD + LBD (*N* = 5), LBD (*N* = 4), FTLD-Tau (*N* = 5), FTLD-TDP (*N* = 5), and FTLD-FUS (*N* = 5) (cohorts drawn from the larger set of cases outlined in supplementary Table 1) were stained with Congo red according to Bely and Makovitzky [10], with additional modifications. Briefly, tissue sections were deparaffinized and dehydrated to dH_2_O, counterstained with hematoxylin (Leica Biosystems, Cat. No. RE7290-K) for 2 min, rinsed in tap water, then stained in 1% fresh aqueous Congo red solution (Sigma-Aldrich, Cat. No. C6767) for 1 h, rinsed in dH_2_O and coverslipped with AquaMounter (BioSB, Cat. No. BSB 0091).

### Assessment of nucleolar *cavities* and the frequency of amyloid aggregates

**Fig. 1:**
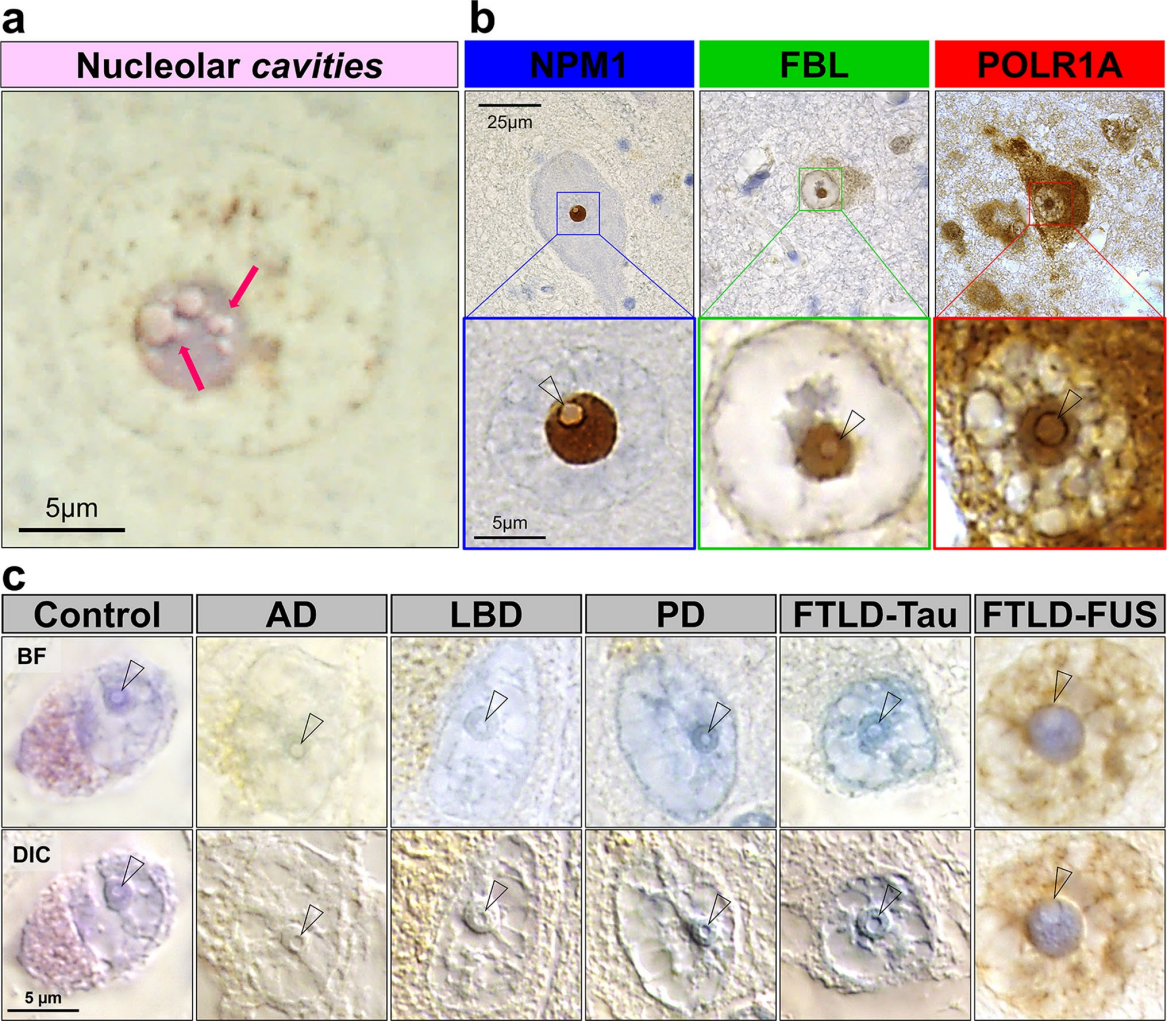
Nucleolar *cavities* that do not stain for nucleolar subcompartment markers can be observed in many neurodegenerative diseases. Formalin-fixed paraffin-embedded brain tissue counterstained with hematoxylin (general DNA and RNA stain) allowed clear observation of neuronal nucleolar *cavities*. Different sizes of *cavities*, as well as fusing events (pink arrows), can be observed within the same nucleolus (**a**). Brain tissue sections immunostained with antibodies against known markers of nucleolar subcompartments—nucleophosmin (NPM1) for the granular component (blue panel), fibrillarin (FBL) for the dense fibrillar component (green panel), and RNA polymerase I subunit RPA194 (POLR1A) for the fibrillar center (red panel)—show that nucleolar subcompartment markers either lightly stain or are excluded from nucleoli *cavities* (open arrowheads) (**b**). Prominent nucleolar *cavities* (open arrowheads) are observed in neurons from aged controls and several neurodegenerative diseases (**c**). Representative brightfield (BF) images in **b** were acquired from FTLD-TDP cases. BF and differential interference contrast (DIC) images in **c** were obtained from temporal cortex sections immunostained with an antibody against the RNA-binding protein CELF1 (CUGBP Elav-Like Family Member 1) from a control case and from patients with Alzheimer’s disease (AD), Lewy body dementia (LBD), Parkinson's disease (PD), FTLD-tau, and from FTLD-FUS patients. All BF and DIC images were acquired with ×100 objective

Nucleolar *cavities* were defined as unstained nucleolar spaces in sections stained for hematoxylin stained (identifies DNA and RNA) or nucleolar proteins (see supplementary Table 3), and classified into (i) unstained *cavities* composed of multiple smaller foci, (ii) a central, large unstained *cavity*, or (iii) a combination of both unstained *cavity* types (Fig. 1).

**Fig. 2:**
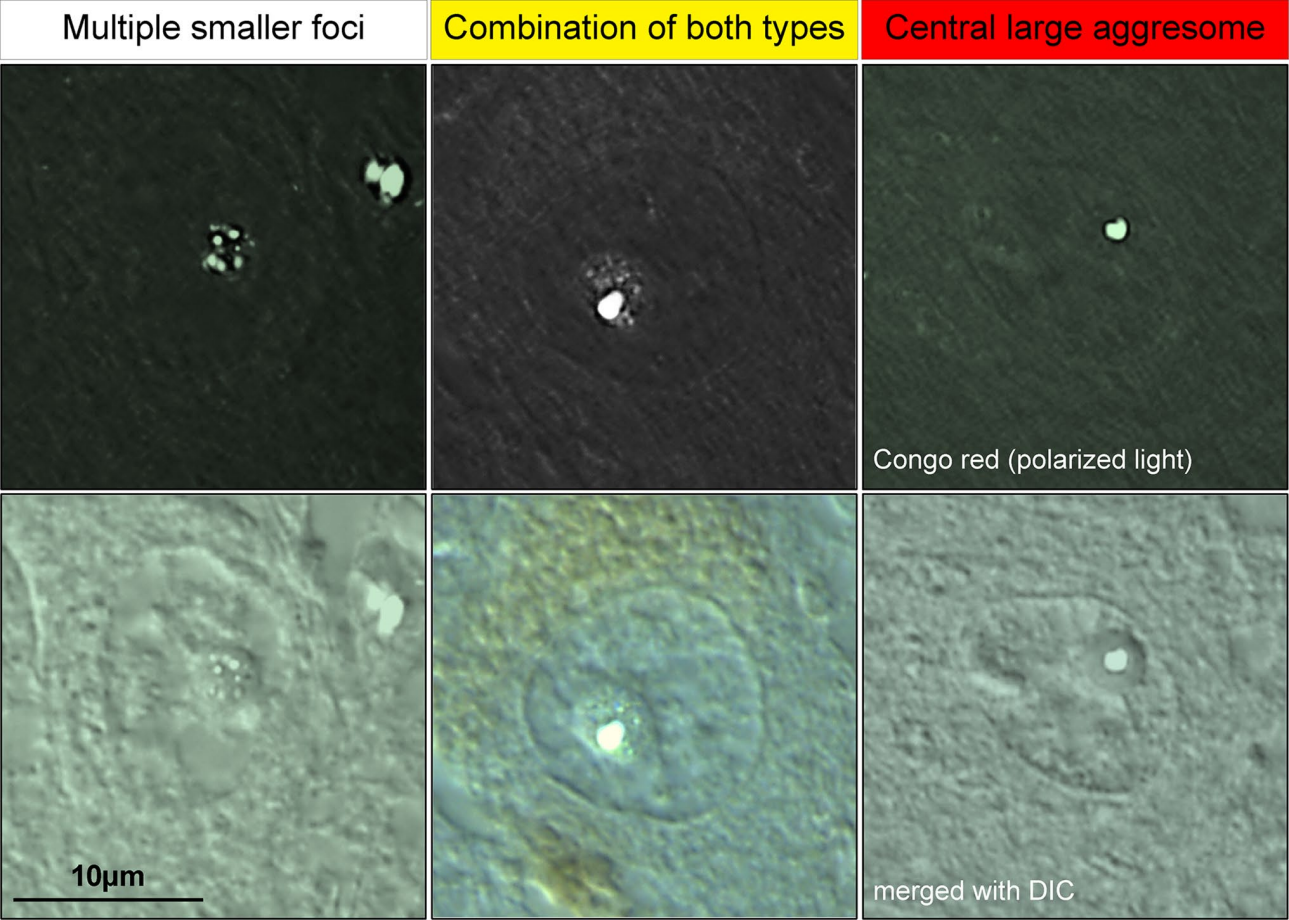
Types of amyloid nucleolar aggregation in postmortem brain tissue. Using Congo red staining in formalin-fixed paraffin-embedded brain tissue, three types of nucleolar aggregates were identified—*multiple smaller foci* are distributed within the nucleolus (left panel); a *central, large aggregate* is a single, large nucleolar aggresome commonly located to the middle of the nucleolus (right panel); and a *combination of both types* presents with small foci distributed more toward the periphery of the nucleolus with a large aggregate more centralized (central panel). These three types are speculated to be part of a continuum leading to the formation of immobilized aggresomes. Representative images were obtained from an aged control and a LATE case using brightfield microscopy with polarizer filter (top row) and DIC (bottom row) with ×100 magnification

Quantification of the presence and type of nucleolar aggregates within nucleolar *cavities* were assessed in tissue sections stained with Congo red (*N* = 43) using a Leica DM6000 upright microscope (Fig. 2). Each slide was observed using the 10× objective (HC PL APO 10×/0.40 DRY) to identify the gray matter. Three regions of interest (ROIs) from the deep cortical layers were randomly selected and marked using Leica LAS X software. The objective was then switched to 100x (HC PL APO 100×/1.40 Oil) to search for nucleolar aggregates in each ROI. First, brightfield microscopy was used to identify a neuron with clearly distinguishable nucleus and nucleolus. The polarizer filter was then applied to search for the presence of amyloid deposits within the nucleolus under polarized light. Each nucleolus was scored as having (1) *no nucleolar cavity or aggregate*; and nucleolar *cavities* with either (2) *several smaller foci*; (3) *a combination of both types*; or (4) *a central large aggresome*. The polarizer filter was removed before searching for the next neuron. For each ROI, 20 neurons were examined, and a total of 2898 neurons were examined for this study.

### Assessment of nucleolar sequestration of neuropathological proteins

Each diagnostic tissue section available for this study was first scanned at low magnification to generate an overview image of the entire section. Three circular regions of interest (ROI1, ROI2, and ROI3), each with a diameter of 1 mm, were separately and randomly placed within the deep layers of the cerebral cortex. These ROIs were then thoroughly examined under 100× objective lenses on either a Leica DM6000 (HC PL APO 100×/1.40 Oil) or Leica DM5000B (HCX PL Fluotar 100×/1.30 Oil) microscope to assess the presence and type of nucleolar aggregation of neuropathological proteins. Within each ROI, the presence of neurons carrying a particular nucleolar aggregation type—central, large aggresome of neuropathological protein; multiple small nucleolar foci of the protein; and a combination of both types—was recorded individually. A “+” was assigned for each nucleolar aggregate type observed within the ROI. After all three ROIs were examined, each aggregation type received a summary score reflecting its distribution across the ROIs: an empty field indicated that the aggregation type was not observed in any ROI; “+” indicated that neurons with this type of nucleolar aggregate were observed in one ROI; “++” indicated the presence in two ROIs; and “+++” indicated the presence of this type of nucleolar aggregate in all three ROIs. The total count across the three ROIs was used to define the overall aggregation profile of each case.

For the *staging* analysis, each case was categorized according to the most advanced type of nucleolar protein aggregation observed, regardless of how many ROIs it appeared in. Representative images, using brightfield and differential interference contrast (DIC) techniques, were acquired under 100× magnification lenses from microscopes Leica DM5000B (HCX PL Fluotar 100×/1.30 Oil), Leica DM6000 (HC PL APO 100×/1.40 Oil), and Nikon Ni-E (Plan Apo Lambda 100×/1.45 Oil).

For *c9orf72* repeat expansions, a separate cohort of cases with poly(GA) inclusions was assessed (*c9orf72*-related FTLD (*N* = 17) and *c9orf72*-related ALS cases (*N* = 11), outlined in supplementary Table 2) to determine the sequestration of any neuropathological proteins. For this analysis, cerebellar sections were evaluated as this region has been shown to reliably identify pathological poly(GA) inclusions in cases with *c9orf72* repeat expansions [15]. The nucleolus of each poly(GA)-positive Purkinje cell was examined for the presence and type of nucleolar *cavities*, as defined above, and whether such *cavities* contained any of the screened pathological proteins. Regardless, all nucleolar *cavities* were classified for each case using the same scoring framework.

### Dichotomous assessment of the impact of nucleolar protein aggregates on the quantity of DNA and rRNA using immunofluorescence

FFPE 10-μm sections of temporal cortex from three aged controls harboring only α-synuclein-positive nucleolar aggresomes and three AD cases harboring only pTau-positive nucleolar aggresomes were deparaffinized and dehydrated to dH_2_O, followed by heat-induced epitope retrieval pretreatment with a pH 6.0 citrate buffer. This analysis was to determine how specific types of nucleolar amyloid aggregates impacted on downstream DNA and rRNA amounts using rRNA immunofluorescence and DAPI fluorescence intensities combined with Congo red staining for amyloids.

Sections were blocked with the Novocastra Protein Block (Leica Biosystems, Cat. No. RE7102) and incubated overnight at 4 °C with a primary anti-rRNA (Y10b) antibody (as detailed in supplementary Table 3). After three washes in 1 × phosphate-buffered saline (PBS) buffer, sections were treated with TrueBlack^®^ Lipofuscin Autofluorescence Quencher (Cell Signaling Technology, Cat. No. 92401) according to the manufacturer’s instructions. Slides were then incubated for 2 h at room temperature with Alexa Fluor™ 488 anti-mouse secondary antibody (Thermo Fisher Scientific, Cat. No. A-21202) diluted 1:150, combined with DAPI 10 µg/mL (Thermo Fisher Scientific, Cat. No. 62248). Following two washes in 1× PBS and one in dH_2_O, sections were stained in freshly prepared 1% aqueous Congo red solution (Sigma-Aldrich, Cat. No. C6767) for 1 h, rinsed in dH_2_O, and coverslipped with ProLong™ Diamond Antifade Mountant (Thermo Fisher Scientific, Cat. No. P36961). Fluorescence images as well as images of Congo red-positive inclusions (acquired under brightfield with polarizing filter) were obtained on a Leica DM6000 upright microscope. For fluorescence channels, acquisition parameters (exposure time, gain, illumination intensity, and filter sets) were kept constant across all sections to ensure comparability of fluorescence intensity. Images were exported as high-resolution 16-bit greyscale TIFF files in greyscale format.

rRNA immunofluorescence and DAPI fluorescence were quantified in Fiji (ImageJ v2.9.0). The pixel-to-micron scale was calibrated from microscope metadata (0.0586 µm/pixel) using the Set Scale function. For each image, uneven illumination was corrected by applying a rolling-ball background subtraction (radius = 100 pixels) uniformly to the entire image. Neuronal somata were then manually delineated as ROIs using morphological criteria (size, visible nucleolus, and DAPI-stained nucleus). For each ROI, the mean gray value and area (µm^2^) were recorded with the Measure function. The mean gray value (in arbitrary units, a.u.) from this pre-processed image was used as the fluorescence readout. To account for any nonspecific background signal, a second ROI was drawn in an adjacent area devoid of cellular profiles or specific fluorescent labeling. The background mean gray value was subtracted from the corresponding neuronal soma ROI value (Mean_ROI - Mean_Background) to yield the final corrected fluorescence intensity for each neuron soma. The corrected values were grouped by diagnostic group and aggresome status for statistical analysis as described below.

### Statistical analysis

Statistical analysis was conducted using the IBM SPSS Statistics software (SPSS Inc., Chicago, IL, USA, version 28). To investigate the differences in nucleolar sequestration among different cohorts, analysis of variance (ANOVA) or Kruskal–Wallis with post hoc protected *t *tests and crosstabs chi-square analysis were used. Spearman correlations were performed to identify significant associations between the stage of nucleolar deposition, *A* scores, *B* scores, *C* scores, Braak LB scores, and Broe staging scores. Age, sex, disease duration, and postmortem delay were used as covariates, and *p *values < 0.05 were considered statistically significant. Graphs were generated using GraphPad Prism (GraphPad Software, San Diego, CA, USA, version 9.5.0).

## Results

### Nucleolar *cavities* are seen in neuronal nucleoli in postmortem human brain

We observed prominent spherical clearings in nucleoli from human brain tissue (Fig. 1a), and further interrogated the neuronal nucleolar environment in FFPE tissue using well established markers for each of the nucleolar subcompartments [46] and hematoxylin staining for DNA and RNA which concentrate in neuronal nucleoli. We found that the spherical clearings do not substantially stain for any nucleolar markers (Fig. 1b), a hallmark of separate structures known as nucleolar *cavities*, previously known as nucleolar *detention centers*. These *cavities*, surrounded by those expected nucleolar subcompartments, are consistent with nucleolar aggregates that sequester proteins undergoing liquid–solid phase transition [37, 49, 50]. Further examination revealed that the distinguishable nucleolar *cavities* were also extensively present in neurons from controls and all neurodegenerative diseases (Fig. 1c), but it was unclear whether they represented a pathophysiology-associated phenomenon. Therefore, we next sought to investigate the nature and frequency of nucleolar *cavities* in the brain of patients with neurodegenerative diseases (cohorts drawn from the larger set of cases outlined in supplementary Table 1).

### Nucleolar *cavities* contain amyloid aggregates that can progress to large aggresomes

Studies have shown that nucleolar *cavities* contain proteins with amyloid characteristics and therefore can be positively stained with amyloidophilic dyes [96]. Using Congo red staining in postmortem brain sections from the temporal cortex from aged controls (*N* = 5) and a variety of neurodegenerative diseases—LATE (*N* = 5), pure AD (*N* = 4), AD + LATE (*N* = 5), AD + LBD (*N* = 5), pure LBD (*N* = 5), FTLD-Tau (*N* = 5), FTLD-TDP (*N* = 5), and FTLD-FUS (*N* = 5) (cohorts drawn from the larger set of cases outlined in supplementary Table 1)—we confirmed the amyloid nature of neuronal nucleolar aggregates observed in all neurodegenerative diseases examined as well as in aged controls (only one had no age-related pathologies). In addition, the neuronal nucleolar aggregates were resistant to proteolytic degradation by proteinase K, pepsin, and trypsin (supplementary Fig. 1), providing strong evidence that these are stable, insoluble structures, formed by proteins with amyloid-like characteristics.

We detected three different presentations of amyloid nucleolar aggregates—one comprised of multiple smaller foci dispersed within the nucleolus, one formed by a single, large aggresome at the center of the nucleolus, and the other was a combination of these two types (Fig. 2). Based on the working model of nucleolar amyloidogenesis described by Wang and colleagues [62], these presentations of nucleolar sequestration are considered part of a continuum where the multiple smaller foci represent the initial steps of amyloidogenesis (either the reversible, liquid phase of A body or the nascent A bodies), the combination of both types represents a physiological state where the nucleolus is heading toward solid-like A bodies, and the central, larger aggregate represents the immobilized, insoluble aggresome. This was confirmed by the observation of fusing unstained nucleolar *cavities* (Fig. 1a), evidence that supports the model proposed [96].

### Nucleolar amyloid aggregates are prevalent but only aggresomes associate with clinical disease

**Fig. 3:**
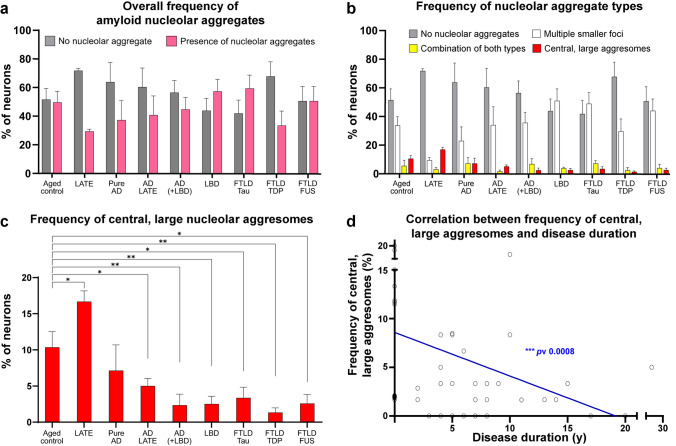
Frequency of neurons with amyloid nucleolar aggregates across several neurodegenerative disorders (means and standard errors displayed. Formalin-fixed paraffin-embedded tissue sections from the temporal cortex stained with Congo red were surveyed for the percentage of neurons with absence (gray bars) or presence (pink bars) of amyloid neuronal nucleolar aggregates (**a**). The presence of amyloid nucleolar aggregates was further assessed by type into *multiple smaller foci* (white bars), *combination of both types* (yellow bars), and *central, large aggresomes* (red bars) (**b**). The frequencies of central, large nucleolar aggresomes were compared using Kruskal–Wallis 1-way ANOVA and a statistically significant variance was observed among the cohorts (*p *value < 0.041) with post hoc pairwise comparisons showing that LATE exhibited a higher frequency of central, large nucleolar aggresomes than aged controls (*p 0.014), whereas other pathological groups showed lower frequencies of central, large nucleolar aggresomes than aged controls—AD + LATE (*p 0.05), AD(+ LBD) (**p 0.004), LBD (**p 0.005), FTLD-Tau (*p 0.019), FTLD-TDP (**p 0.002), and FTLD-FUS (*p 0.026) **(c)**. A statistically significant, negative correlation was detected between the frequency of central, large aggresomes and disease duration (***p value 0.0008) (**d**). Cases were grouped according to their pathological diagnosis into aged controls, limbic-predominant age-related TDP-43 encephalopathy (LATE), AD (Alzheimer’s disease) ± LATE, AD with LBD (Lewy body disease), pure LBD, FTLD-TDP, FTLD-Tau, and FTLD-FUS

We next examined the frequency of neurons containing amyloid nucleolar aggregates. Further analysis revealed that while nucleolar aggregates are present in neurons across all cohorts (including aged controls), their overall frequency varies greatly between conditions. Aged controls and FTLD-FUS showed a relatively balanced proportion of neurons with and without nucleolar aggregates, suggesting that such aggregates occur dynamically with normal aging. LATE, pure AD, AD + LATE, AD + LBD, and FTLD-TDP exhibited a higher proportion of neurons without nucleolar aggregates, whereas LBD, and FTLD-Tau had a higher frequency of neurons carrying nucleolar aggregates (Fig. 3a). We did not observe a clear relationship between their overall presence or absence and specific conditions, which prompted us to investigate whether the different types of nucleolar aggregates might be more closely associated with specific cohorts.

Multiple smaller foci were the most common type of nucleolar aggregates across all groups, except for the LATE cohort. Central, large aggresomes were considerably less frequent but showed higher frequency in the asymptomatic cohorts with age-related pathologies (aged controls and LATE) (Fig. 3b). In fact, there was only a significant difference in the proportion of neurons containing central, large aggresomes among the cohorts (Kruskal–Wallis *p *value < 0.041, for none or other aggregate types all *p *values > 0.05), and post hoc pairwise comparisons revealed significantly different frequencies of nucleolar aggresomes in aged controls compared to LATE (*p *value 0.014), AD + LATE (*p *value 0.050), AD(+ LBD) (*p *value 0.004), LBD (*p *value 0.005), FTLD-Tau (*p *value 0.019), FTLD-TDP (*p *value 0.002), and FTLD-FUS (*p *value 0.026) (Figs. 3c), suggesting that neurons containing nucleolar aggresomes are more vulnerable to neurodegeneration.

Additionally, a significant negative correlation was observed between the frequency of central, large aggresomes and clinical disease duration (*p *value 0.0008) (Fig. 3d), further supporting the hypothesis that neurons containing this aggregate type are more prone to degeneration as disease progresses. No clear relationship or significant correlation was found between the frequency of the different nucleolar aggregate types and clinical or neuropathological variables, such as gender, postmortem delay, Braak staging, LATE TDP-43 stage, or brain weight. Together, our analysis suggests that multiple smaller foci are indicative of a physiological response in nucleoli in the aged, whereas central, large aggresomes represent pathology-associated aggregates. Therefore, we next aimed to investigate the proteins within these nucleolar aggregates, particularly neuropathological proteins known to form amyloid-like aggregates in the neurodegenerative brain.

### Aβ aggregates occur in a small proportion of nucleolar aggresomes but not prior to Aβ plaque deposition

**Fig. 4:**
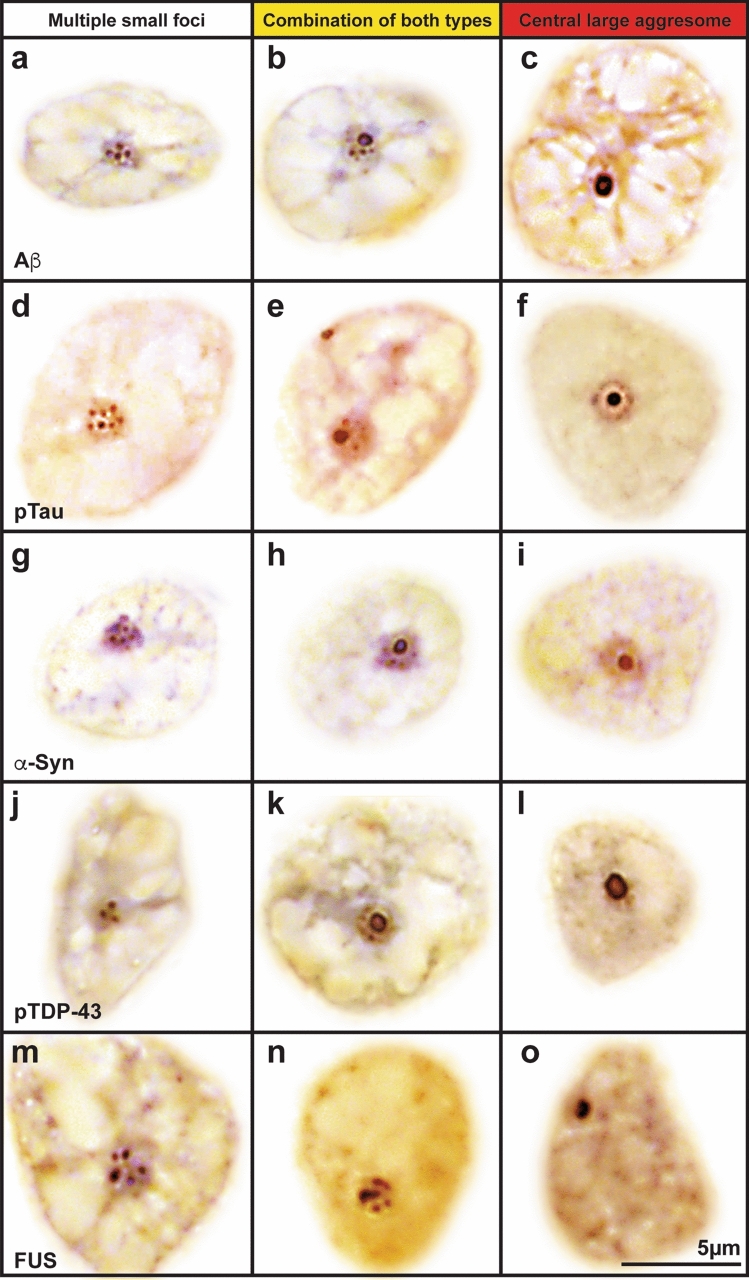
Nucleolar sequestration of key neuropathological proteins in the neurodegenerative brain. FFPE tissue sections from postmortem brain from patients with neurodegenerative diseases were immunostained with antibodies against five neuropathological proteins—Aβ (**a**–**c**), phosphorylated tau (pTau **d**–**f**), α-synuclein (**g**–**i**), phosphorylated TDP-43 (pTDP-43 **j**–**l**), and FUS (**m**–**o**). All neuropathological proteins examined are sequestered into the three identified types of nucleolar aggregates: multiple smaller foci (**a**, **d**, **g**, **j**, **m**), a combination of both types (**b**, **e**, **h**, **k**, **n**), and a central large aggresome (**c**, **f**, **i**, **l**, **o**). Representative images for Aβ were obtained from the frontal cortex of AD cases; images from the anterior cingulate cortex from AD cases were used for both pTau and α-synuclein; for pTDP-43 images were obtained from the superior frontal cortex of FTLD-TDP cases; and for FUS images were obtained from the hippocampus, striatum, and motor cortex of FTLD-FUS cases. All images are from neuronal nuclei where the nucleolus can be visually identified and were acquired using brightfield microscopy with ×100 objective. For this figure, neuronal nuclei were cropped from the surrounding tissue to emphasize nuclear and nucleolar morphology. Color adjustments were applied to remove nucleic acid counterstaining (blue) from RGB. The original images can be seen in supplementary Fig. 2

**Fig. 5:**
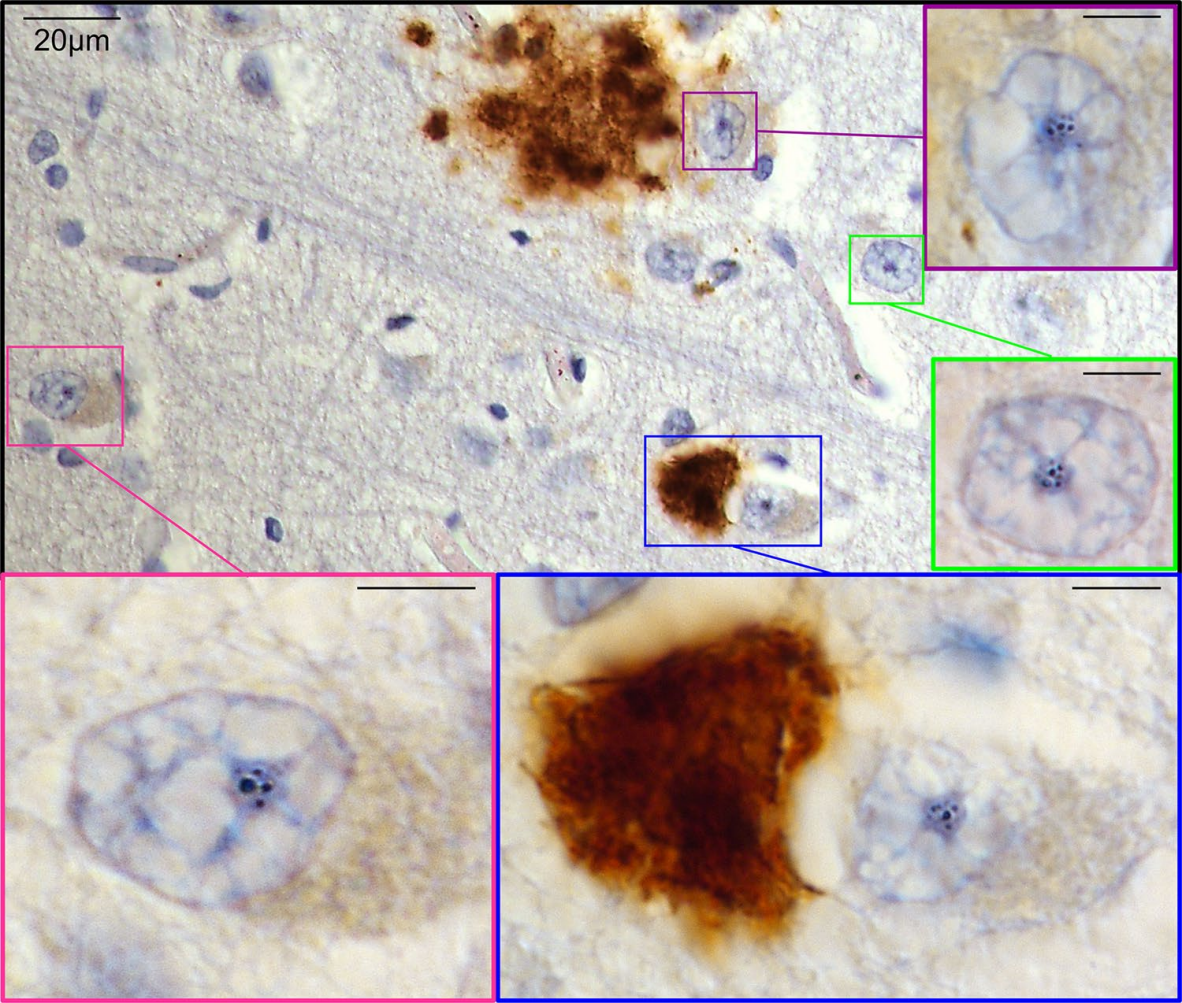
Localization of neurons containing nucleolar Aβ inclusions in relation to Aβ plaques. Neurons carrying nucleolar Aβ foci and/or aggregates are not necessarily in the immediate vicinity of an Aβ plaque. Representative image of a tissue section from the frontal cortex of an AD case showing a neuronal nucleus with multiple smaller Aβ foci in its nucleolus located in very close proximity to a large amyloid plaque (purple inset); a neuronal nucleolus with multiple smaller Aβ foci further to the large Aβ plaque (green inset); a neuronal nucleus with a combination of nucleolar inclusion types next to a smaller Aβ plaque (blue inset); and a neuronal nucleus carrying the combination of both nucleolar aggregation types without any Aβ plaque in its immediate vicinity (pink inset). Images were acquired using brightfield microscopy with ×100 magnification. All scale bars from insets represent 5 µm

Nucleolar Aβ A bodies have been suggested as initiating seeding for Aβ plaques found in AD (for review see [96]). This has been demonstrated in vitro and in cell models [8, 16, 97] but nucleolar Aβ A bodies have not been identified in postmortem brain tissue from AD patients. FFPE tissue from frontal and temporal cortices from nine cohorts (outlined in supplementary Table 1) confirmed that Aβ is sequestered into the three different types of nucleolar aggregates—multiple small Aβ foci, a single, large Aβ aggresome at the center of the nucleolus, or the combination of these two types (Fig. 4a–c). Within the same tissue sections, the position of neurons containing different types of nucleolar inclusions in relation to the Aβ plaque could not be properly established, as these neurons could be found in the immediate vicinity and further away from the Aβ plaques (Fig. 5).

**Fig. 6:**
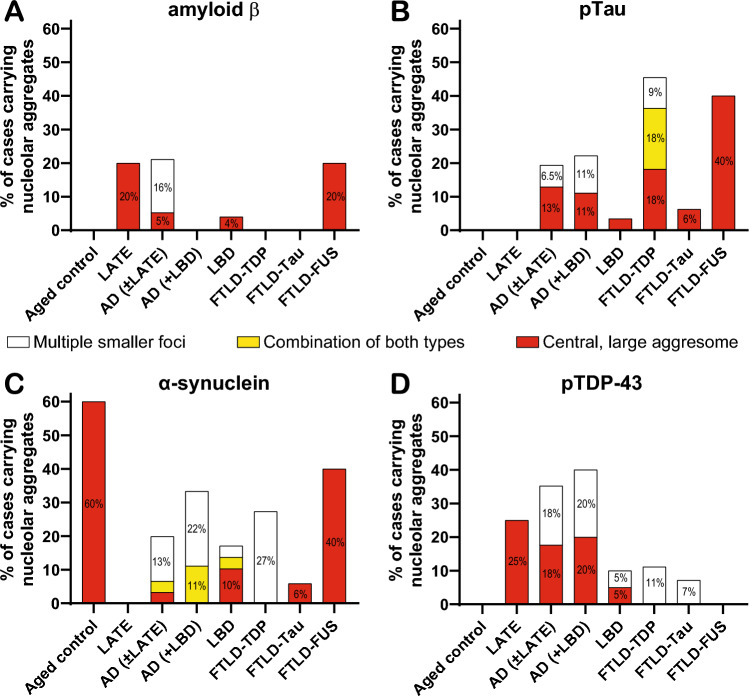
Occurrence of nucleolar sequestration types of neuropathological proteins in the temporal cortex from different cohorts classified according to their pathological diagnosis. Diagnostic formalin-fixed paraffin-embedded tissue sections from the temporal cortex immunostained for four neuropathological proteins—Aβ (**a**), pTau (**b**), α-synuclein (**c**), and pTDP-43 (**d**)—were assessed for the presence of *multiple smaller nucleolar foci* (white bar sections), *combination of both types* of nucleolar sequestration (yellow bar sections), and *central, large nucleolar aggresomes* (red bar sections). Cases were grouped according to their pathological diagnosis into aged controls, limbic-predominant age-related TDP-43 encephalopathy (LATE), AD (Alzheimer’s disease) ± LATE, AD with LBD (Lewy body disease), pure LBD, FTLD-TDP, FTLD-Tau and FTLD-FUS. Graph bars show the percentage of positive cases among each cohort

We examined the temporal cortex and identified nucleolar Aβ aggresomes in a subset of cases from the LATE (20%), AD ± LATE (5%), LBD (4%), and FTLD-FUS (20%) cohorts. Additionally, multiple small Aβ foci were observed in 16% of AD ± LATE cases (Fig. 6a). To assess whether nucleolar Aβ aggregation is associated with the spread of Aβ plaque in the brain, we classified all cases using the *A* score system (A0 to A3), which reflects the phase of amyloid β deposition from none (A0) to widespread cortical involvement (A3) [36]. For this analysis, cases were grouped into *Aβ plaque negative* (A0) and *Aβ plaque-positive* (A1–A3) categories.

**Fig. 7:**
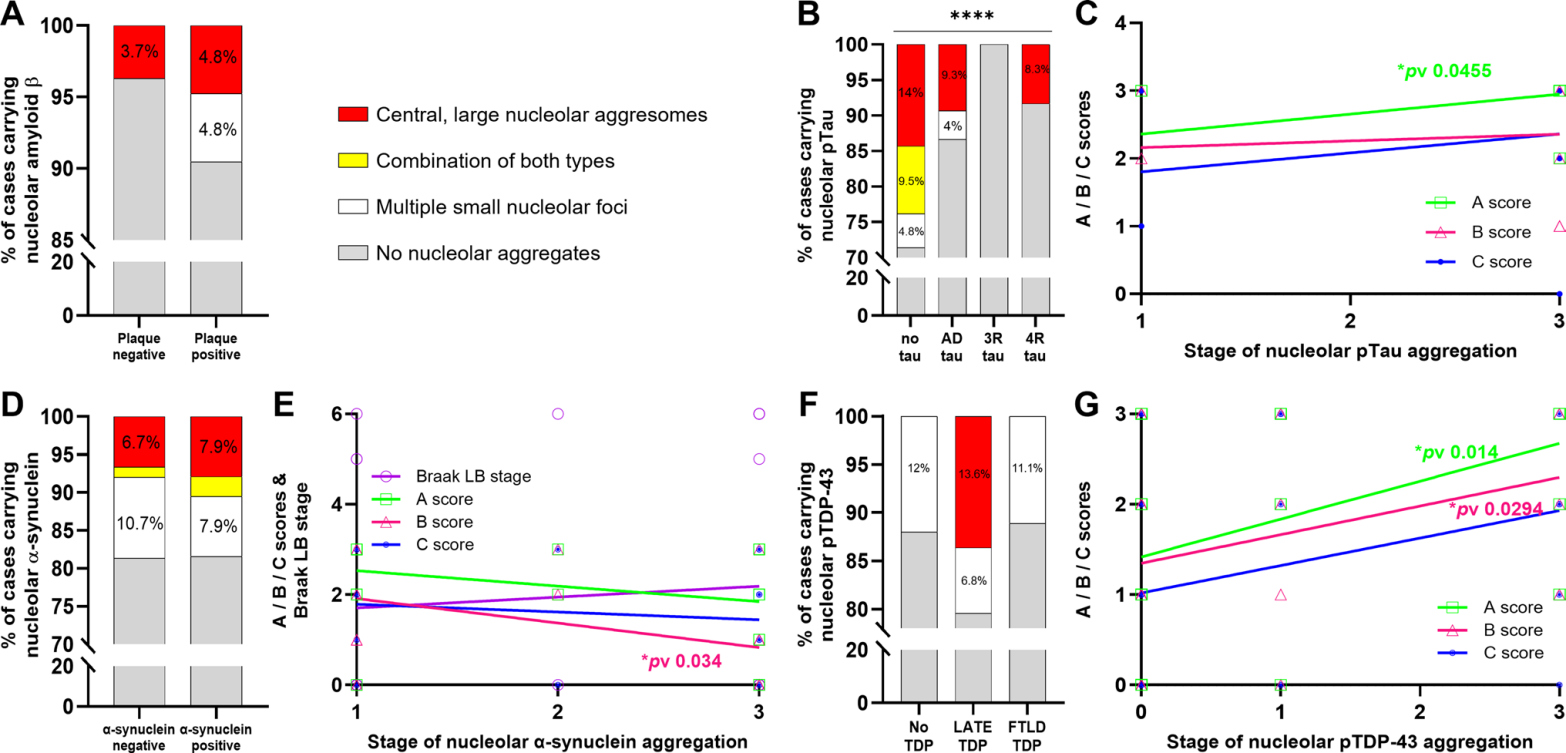
Distribution and correlation of nucleolar sequestration of neuropathological proteins in cohorts classified according to their pathological load. Cases were re-grouped according to their Aβ plaque load (*A* score) into plaque negative (A0 cases) or plaque-positive groups (A1, A2, and A3 cases) with multiple small Aβ foci only present in the plaque-positive group and central, large nucleolar aggresomes present in both groups (**a**). Clinical cases were grouped according to their neurofibrillary tangle stage (*B* score) and their type of tau filament into four groups: no tau (B0 cases), AD tau (cases with AD neuropathic changes and B1, B2, or B3 scores), 3R tau (Pick’s disease), and 4R tau (globular glial tauopathy, progressive supranuclear palsy, and corticobasal degeneration). More advanced stages of nucleolar pTau sequestration were found, in a decreasing manner, in no tau, AD tau, and 4R tau groups. The difference between groups was statistically significant (****pv < 0.0001) (**b**). *A* scores from cases included in the AD tau cohort significantly correlate with the stage of their nucleolar pTau aggregates (*pv 0.0455) (**c**). Cases grouped according to the topographical extent of their pathological lesions (Braak LB score) into two groups: α-synuclein negative cohort (Braak LB score 0) and α-synuclein-positive cohort (Braak LB scores of 4, 5, and 6). Similar distributions of nucleolar α-synuclein aggregate types were found in both groups (**d**). Variables from all cases with nucleolar α-synuclein aggregates were correlated with the stage of their nucleolar α-synuclein aggregates. Neurofibrillary tangle stage (*B* score) negatively correlated with Lewy body stage (*pv 0.034) (**e**). Cases with TDP-43 aggregates were grouped according to their LATE status or FTLD-TDP subtypes into 3 groups: no TDP, LATE TDP (stages 1 and 2), and FTLD-TDP types A, B and C. Multiple small pTDP-43 foci were present in all groups, while nucleolar pTDP-43 aggresomes were only present in LATE TDP group (**f**). A significant, positive correlation was detected between the stages of nucleolar aggregates from all cases carrying nucleolar pTDP-43 aggregates and their *A* and *B* scores (*pv 0.014 and *pv 0.0294, respectively) (**g**)

We found that cases from the *Aβ plaque-positive* cohort had multiple Aβ foci and central, large Aβ aggresomes, whereas the *Aβ plaque negative* cohort only had a single case carrying Aβ aggresomes and no other aggregate types (Fig. 7a). Our findings indicate that, in the temporal cortex, nucleolar Aβ aggregation does not precede extracellular plaque deposition, and therefore do not support the hypothesis proposed [96] that nucleolar Aβ aggregation seeds Aβ plaque formation. Instead, nucleolar Aβ aggregates are most likely a response associated with cellular stress induced by existing Aβ plaques.

### Nucleolar aggregation of phosphorylated tau associates with all clinical neurodegenerative diseases and with increasing amounts of neuropathology

Tau protein has previously been reported to aggregate into stress-inducible A bodies in cell culture models [16]. The occurrence of nucleolar sequestration of phosphorylated tau (pTau) was assessed in FFPE tissue sections from the temporal cortex of the same disease and control cohorts (see supplementary Table 1). We observed the same types of nucleolar pTau bodies as previously found for Aβ—multiple small pTau foci, a central, large aggregate of nucleolar pTau resembling solid-like aggresomes [96], and co-occurrence of both types (Fig. 4d–f). Notably, central, large pTau aggresomes also appeared in neurons carrying cytoplasmic pTau in brains from patients with AD ± LATE and AD + LBD (supplementary Fig. 3).

Despite the presence of age-related sparse neurofibrillary tangles and plaque pathology, aged controls and asymptomatic LATE did not present any type of nucleolar pTau aggregates (Fig. 6b), indicating that, similar to Aβ, nucleolar pTau does not precede the development of Alzheimer-related tangles and Aβ plaques. However, both AD cohorts—AD ± LATE and AD + LBD—presented multiple small nucleolar foci (6.5% and 11%, respectively) and central, large nucleolar pTau aggresomes (13% and 11%, respectively), while LBD, FTLD-Tau and FTLD-FUS cohorts only displayed pTau aggresomes (3.5%, 6% and 40%, respectively). While pTau and α-synuclein pathologies are well known to co-exist [69, 103], nucleolar sequestration of pTau with co-existing LBD pathologies has not been explored previously. The FTLD-TDP cohort displayed a broader distribution, with 9% of cases displaying small, multiple nucleolar foci, 18% presenting pTau aggresomes, and another 18% containing a combination of both types. To the best of our knowledge, this is the first observation of nucleolar tau, phosphorylated or not, in FTLD-TDP (Fig. 6b). The presence of nucleolar pTau aggregates in all disease groups, but not in aged controls or asymptomatic LATE cases, suggests a strong association to neurodegeneration rather than a normal age-related phenomenon. Furthermore, the presence of nucleolar pTau aggresomes across all disease cohorts indicates a convergent pathological mechanism in neurodegeneration, independent of disease classification.

We next assessed whether the isoform type of tau filament impacted on nucleolar pTau accumulation in the clinical disease cohorts using four groups: *AD tau* (AD neuropathic changes and *B* score > B0, indicating the presence of neurofibrillary tangle pathology), *3-repeat tau (3R tau* = Pick’s disease), *4-repeat tau (4R tau* = globular glial tauopathy, progressive supranuclear palsy, and corticobasal degeneration) and *those with no tau inclusions* (cases with *B* score = B0—largely LBD, FTLD-TDP and FTLD-FUS cases). Clinical cases without neurofibrillary tangles (*no tau* cohort) had all three types of nucleolar pTau aggregates—multiple small foci (4.8%), combination of both types (9.5%) and pTau aggresomes (14%)—whereas *AD tau* and *4R tau* cohorts display smaller proportions of nucleolar pTau foci (in *AD tau* only, 4%) and aggresomes (9.3% and 8.3%, respectively). Cases from the *3R tau* cohort did not present any type of nucleolar pTau aggregation. Pearson’s chi-square test found statistically significant differences in the overall presence of nucleolar pTau aggregates between the four cohorts (Fig. 7b). The presence of pTau aggregates, especially aggresomes, in the *no tau* cohort suggests that those structures may precede, and potentially seed, tau pathology in cases with other diagnostic pathologies. Furthermore, the lower prevalence of nucleolar pTau aggregates, particularly aggresomes, in the tau cohorts may also suggest that neurons containing these structures were more vulnerable to cell death, reducing the presence of such nucleolar pTau aggregates.

Interestingly, in the *no tau* cohort, 33% of cases carrying any type of pTau aggregate have a positive *A* score, while in the *AD tau* cohort, 100% of cases carrying nucleolar pTau aggregates have a positive *A* score (with 90% having *A* score = 3). When staging the *AD tau* cases as 0–3 depending on the absence (0) or the most advanced type of nucleolar aggregate observed in the tissue (1 = the presence of multiple small nucleolar foci, 2 = a mix of small foci and larger aggregates, and 3 = having large, central nucleolar aggresomes), there was a significant positive correlation between the advancement of pTau nucleolar aggregates and the burden of Aβ plaque (*A* score, *p *value 0.0455) (Fig. 7c). Together these data show an association between the presence of nucleolar pTau aggresomes and significant neuronal and Aβ plaque pathologies in cases with clinical neurodegenerative diseases.

### Neuronal nucleolar α-synuclein aggresomes appear protective and dissipate in the presence of tau pathology

A recent study reported the presence of α-synuclein within the nucleolus of a cancer cell line, where it regulates ribosomal DNA damage repair [7]. However, α-synuclein has never been observed as nucleolar aggresomes, despite its amyloid-like aggregation-prone domain [57] and its capacity to aggregate into stress-inducible A bodies [16]. We therefore assessed whether α-synuclein is also sequestered in the nucleolus in FFPE tissue sections from the temporal cortex in the same cohorts (see supplementary Table 1). We observed the same types of nucleolar structures as those noted for Aβ and pTau (Fig. 4g–i) using an anti-pan α-synuclein antibody [42/α-Synuclein] with more nucleolar sequestration found in pure LBD cases using an anti-phosphorylated α-synuclein antibody [EP1536Y] (supplementary Fig. 4).

A great proportion of aged controls (60%) had centralized, large nucleolar α-synuclein aggresomes and no other type of aggregates, whereas asymptomatic LATE subjects did not present any type of nucleolar α-synuclein aggregate (Fig. 6c). The identification of nucleolar α-synuclein aggresomes in aged controls, in the absence of neurodegeneration, suggests that these structures are not intrinsically harmful and may play a protective or compensatory role, as recently shown [7]. Among disease cohorts, LBD and AD ± LATE were the only other groups to exhibit nucleolar α-synuclein aggregate types with more central, large α-synuclein aggresomes in LBD (10% and 3%, respectively) and fewer multiple small foci compared to AD (3% and 13%, respectively). The AD + LBD cohort had 22% of cases with multiple small foci, while 11% showed a combination of both types, and the FTLD-TDP cohort only presented cases with multiple small nucleolar α-synuclein foci (27%). FTLD-Tau and FTLD-FUS cohorts only carried central, large nucleolar α-synuclein aggresomes (Fig. 6c). These data suggest that controls, LBD, FTLD-Tau and FTLD-FUS have nucleolar α-synuclein aggresomes potentially to repair DNA.

We next investigated the relationship between nucleolar α-synuclein and the spread of α-synuclein Lewy pathology in the brain [using Braak Lewy body (LB) score—stages 4, 5, and 6 [12]]. The three types of nucleolar α-synuclein aggregates were found in similar proportions in both *α-synuclein negative* (Braak LB score negative) and *α-synuclein-positive* [Braak LB score positive (stage 4–6)] groups (Fig. 7d), suggesting that nucleolar aggregation of α-synuclein does not contribute to the seeding of Lewy body pathology. This may have been expected considering that most groups without Lewy pathology had nucleolar α-synuclein aggregates (Fig. 6c). Not surprisingly, Pearson’s chi-square tests found no difference in the presence of neuronal nucleolar α-synuclein between the groups, or the individual types of nucleolar α-synuclein aggregates (Fig. 7d).

Similar to pTau, we also investigated the factors contributing to the stage transition of nucleolar α-synuclein aggregates in the temporal cortex. While there was no correlation with any other clinical or neuropathological variable, a significant negative correlation was observed between the advancement of nucleolar α-synuclein aggregation and Braak tangle staging (*B* score, *p* value 0.034) (Fig. 7e), indicating that cases with a higher neurofibrillary tangle burden (high *B* score) were less likely to exhibit α-synuclein aggresomes. The observed negative correlation with *B* score suggests that as tau pathology progresses, neurons reduce their α-synuclein aggresomes as a consequence of a broader stress response triggered by tau pathology.

Collectively, our study suggests that the assembling and progression of nucleolar α-synuclein aggregates may be protective, and that these nucleolar inclusions dissipate when tau pathology occurs and advances. Therefore, although our findings do not support a role for nucleolar α-synuclein aggregates in seeding Lewy body pathology, they do establish a complex interplay between nucleolar α-synuclein aggregation and tau pathology.

### Nucleolar pTDP-43 aggresomes are a pathological feature of LATE pathology

Although nucleolar stress has been implicated in TDP-43 pathology [4, 5, 67], the aggregation-prone, phosphorylated TDP-43 (pTDP-43) has never been reported in the nucleolus. We performed the same analysis as for the previously assessed pathological proteins in the temporal cortex of the same cohorts (see supplementary Table 1). pTDP-43 was found in nucleolar aggregates mostly as multiple small foci or large central aggresomes, and rarely aggregates of both types (Fig. 4j–l). Aged controls and FTLD-FUS cases did not present any type of nucleolar pTDP-43 aggregates (Fig. 6d). In contrast, 25% of asymptomatic LATE, 18% of AD ± LATE, 20% of AD + LBD, and 5% of LBD cases had central, large pTDP-43 aggresomes. A proportion of AD ± LATE, AD + LBD, and LBD cohorts also showed multiple small foci (18%, 20%, and 5%, respectively). FTLD-TDP and FTLD-Tau cohorts only had multiple small pTDP-43 foci (11% and 7%, respectively) (Fig. 6d). The presence of nucleolar pTDP-43 aggregates in non-TDP-43 proteinopathies suggests that these aggregates do not seed TDP-43 pathology. Additionally, the presence of nucleolar pTDP-43 aggresomes in the LATE and AD ± LATE but not in the FTLD-TDP cohort, as well as their presence in the AD + LBD and LBD cohorts but not in FTLD-Tau cases, suggests that nucleolar sequestration of pTDP-43 into aggresomes may emerge from a combined pathological burden.

To further investigate nucleolar pTDP-43 aggregation, we reorganized cases into three groups based on TDP-43 pathology: no TDP, LATE TDP (stages 1&2), and FTLD-TDP. All groups exhibited similar proportions of multiple small pTDP-43 foci, but central, large pTDP-43 aggresomes were exclusive to the LATE TDP group (Fig. 7f). This observation highlights nucleolar sequestration of pTDP-43 into aggresomes as a novel pathological feature strongly associated with LATE pathology.

As with the other proteins, we investigated the factors that may determine the stage of nucleolar pTDP-43 aggregates by correlating the aggregation stages with clinical and neuropathological variables. We found that the advancing stages of pTDP-43 aggregates correlated positively with Aβ plaque burden (*A* score, *p *value 0.014) and with the spread of neurofibrillary tangles (*B* score, *p *value 0.0294) (Fig. 7g), suggesting that higher A and B pathological scores are more likely to be associated with neurons containing pTDP-43 aggresomes. In fact, the LATE TDP group exhibited a higher proportion of cases with positive *A* scores (77.3%), *B* scores (95.5%), and *C* scores (59.1%) compared to the FTLD-TDP group (44.4%, 44.4%, and 22.2%, respectively).

This difference in pathological burden may help explain why nucleolar pTDP-43 aggregates in the LATE TDP group transitioned to aggresomes, whereas they did not in FTLD-TDP cases. Increasing Aβ and tau pathologies in LATE TDP cases may play a significant role by promoting the progression of pTDP-43 aggregates into solid-like aggresomes or by inducing nucleolar stress that facilitates their accumulation. Our findings suggest that nucleolar aggregation of pTDP-43 does not seed TDP-43 pathology, but strongly associates with LATE pathology in the presence of Aβ and tau pathologies.

### FUS is only rarely sequestered into nucleolar aggregates

The fused in sarcoma (FUS) protein—another fibrillation-prone, neurodegeneration-related protein [60]—is diffusely located in the nucleolus upon stress in cell models [64, 106]. As this substantially differs from the aggregate-like presentations observed in brain tissue for other neuropathological proteins, we decided to assess the nucleolar sequestration of FUS in the same cohorts (see supplementary Table 1). Since FUS is part of the FET protein family, the other two members of this family—EWS and TAF15—were also examined. Unlike the other fibrillation-prone proteins in this study, we did not observe neuronal nucleolar sequestration of FUS, EWS, or TAF15 in the temporal cortex of any cohort analyzed. However, when we analyzed additional sections from the frontal and anterior cingulate cortices we identified multiple smaller FUS foci, central, large FUS aggresomes, and a combination of both types, but only in a few FTLD-FUS cases (Fig. 4m–o). This suggests that FUS nucleolar sequestration is relatively rare compared with the other fibrillation-prone proteins.

### No nucleolar sequestration of other neurodegeneration-associated, aggregation-prone proteins

#### Prion protein

The cellular prion protein (PrP^C^), encoded by the *PRNP* gene, participates in several biological processes, including neuronal protection and homeostasis, neurogenesis, synaptic modulation, cell adhesion and signaling, and copper metabolism [56]. Mutations within exon 3 of the *PRNP* gene or the pathological conversion of natively folded PrP^C^ into its misfolded and infectious prion form (PrP^Sc^) lead to progressive, fatal neurodegenerative prion diseases [70]. Despite its spontaneous propensity to aggregate into structurally distinct amyloid fibrils and plaques [101, 105], our histological investigations did not detect any occurrence of nucleolar aggregation of PrP^C^ in the frontal, temporal, or anterior cingulate cortices in cases from all cohorts previously examined (see supplementary Table 1) although distinct patterns of staining for the protein were observed (supplementary Fig. 5).

#### Poly(GA) dipeptide repeat protein

Dipeptide repeat proteins (DPR) are produced by RAN translation from sense and antisense transcripts of hexanucleotide repeat expansions in the *c9orf72* gene [19]. Particularly, poly(GR) and poly(PR) have been found in nucleoli from cell models, affecting the GC phase-separation properties and disrupting the nucleolar function of reversibly storing misfolded proteins [27, 28, 45, 52, 99]. To investigate a possible relationship between DPR and nucleolar *cavities* in postmortem human brain we used FFPE tissue sections from the cerebellum from *c9orf72*-related FTLD-TDP (*c9*FTLD, *N* = 17) and *c9orf72*-related ALS patients (*c9*ALS, *N* = 11) (see supplementary Table 2), immunostained with an antibody against poly(GA)—the most toxic and aggregation-prone DPR [27, 53]—as well as with pTDP-43 and pTau antibodies. We focused our analysis on Purkinje cells due to their large cell body with a distinguishable nucleolus, features enabling proper nucleolar investigations.

**Fig. 8:**
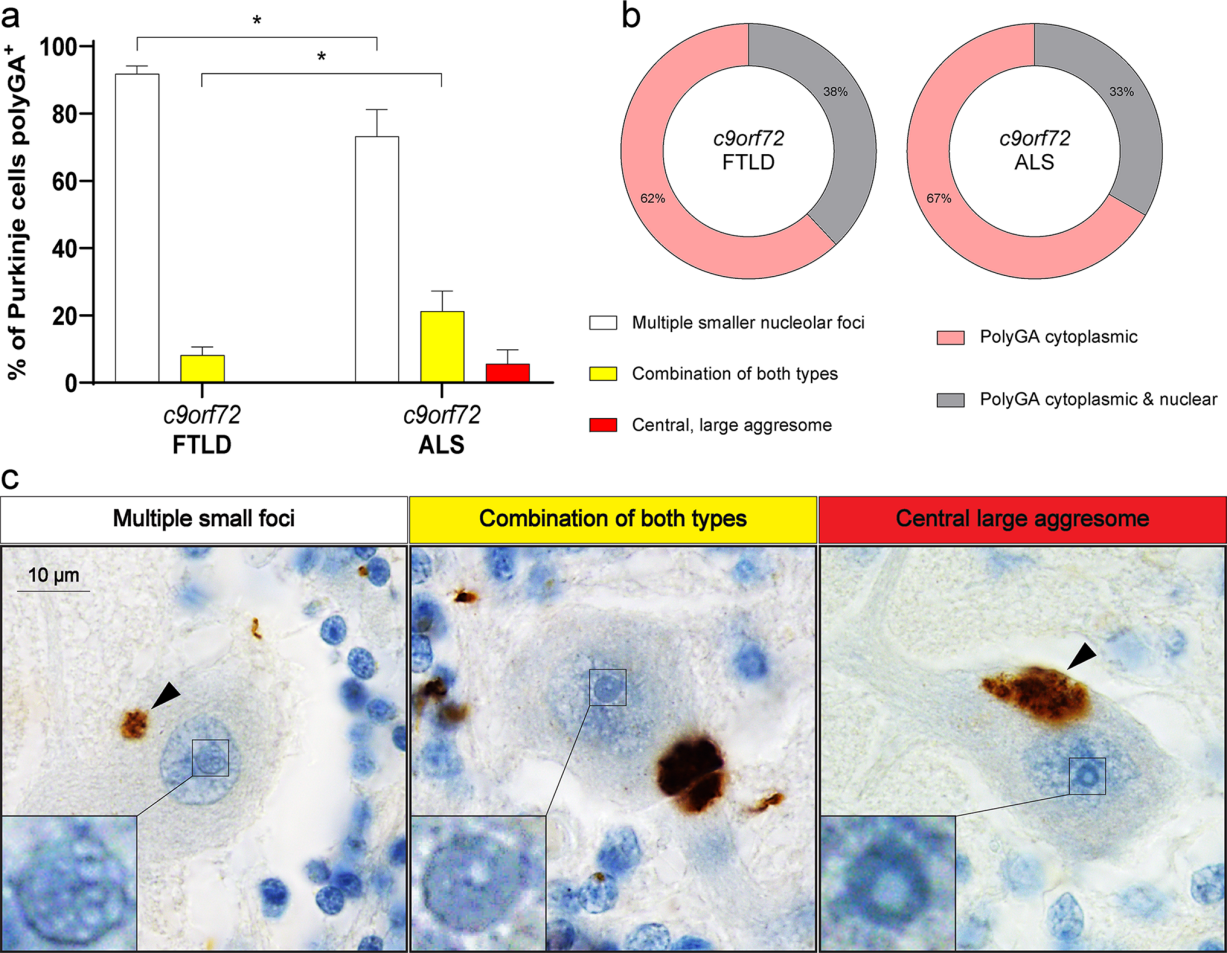
Purkinje cells carrying poly(GA) inclusions also display nucleolar *cavities*. Distribution (mean and standard errors) of nucleolar *cavity* types in Purkinje cells carrying poly(GA) inclusions in *c9orf72*-related FTLD and *c9orf72*-related ALS (*pv < 0.05) (**a**). Percentage of Purkinje cells carrying cytoplasmic versus nuclear poly(GA) inclusions in *c9orf72*-related FTLD and *c9orf72*-related ALS (**b**). Representative images of types of nucleolar *cavities* (insets) within Purkinje cells carrying cytoplasmic poly(GA) (arrowheads) were obtained from the cerebellum of FTLD patients (showing *cavities* associated to multiple smaller foci and combination of both types) and an ALS patient (showing a *cavity* associated to a central large aggresome) (**c**). Images were acquired using brightfield microscopy with ×100 objective lens

In line with the model presented by Frottin and colleagues [28], we noted the presence of amyloid-like aggregation of poly(GA) in the nucleoplasm of those neurons, as well as in the cytoplasm as reported by other researchers [104], but not inside the nucleolus. Importantly, however, we found that poly(GA) inclusions are exclusively present in Purkinje cells whose nucleoli carry prominent nucleolar *cavities*. The percentage of poly(GA)^+^ Purkinje cells displaying clearings compatible with multiple smaller foci within the nucleolus in *c9*FTLD is significantly higher than those in *c9*ALS (*p value 0.0176), and the percentage of cells carrying a combination of both *cavity* types in *c9*FTLD is significantly lower than those in *c9*ALS (*p value 0.0346). Central large *cavities*, compatible with nucleolar aggresomes, were only observed in *c9*ALS (Fig. 8a). The significant differences between *c9orf72* FTLD and *c9orf72* ALS might be explained by the fact that, interestingly, all Purkinje cells with nuclear poly(GA) inclusions exclusively display multiple smaller foci of nucleolar clearings. Given that *c9orf72* FTLD Purkinje cells carry slightly less cytoplasmic poly(GA) and slightly more nuclear poly(GA) than *c9orf72* ALS cases (Fig. 8b), it is expected that, overall, *c9orf72* FTLD poly(GA)^+^ neurons will have more *cavities* than *c9orf72* ALS. It is worth noting that most nucleolar *cavities* corresponding to multiple ‘smaller’ foci in the poly(GA)^+^ Purkinje cells seem larger as they take over most of the nucleolar environment (Fig. 8c), but the nucleoli in these cases are smaller in size [4]. In an effort to identify which proteins aggregate within the nucleolus of *c9*FTLD and *c9*ALS cerebellum, we extended our investigation to assess the potential nucleolar aggregation of pTDP-43 and pTau in the same cohort. This analysis however did not identify any instances of nucleolar pTDP-43 or pTau in Purkinje cells across all cases examined, leaving the protein composition of these nucleolar aggregates yet to be determined.

#### PolyQ tract

Huntington’s disease (HD), a genetic neurodegenerative condition characterized by progressive choreiform movements and cognitive and behavioral symptoms, is caused by the presence of > 35 CAG trinucleotide repeats within the first exon of the *huntingtin* gene [1]. This translates into an abnormally long polyglutamine expansion (polyQ tract) in the N-terminal of the huntingtin protein (HTT), ultimately leading to protein misfolding, aggregation, and toxicity [35]. Normal HTT is diffusely present in the nucleolus of human fibroblasts, suggesting a role in rRNA processing or ribosome biogenesis [41]. Differently, polyQ-HTT forms intranuclear and cytoplasmic deposits, with structural and biochemical differences between the two [79]. A mutated form of HTT was diffusely located in the nucleolus of a HD cell line [93] though, to date, the pathological nucleolar aggregation of the polyQ-HTT has not been reported. Since nucleolar dysfunction is a key driver of HD pathogenesis [51, 94], we examined FFPE sections from the frontal, temporal, and anterior cingulate cortices, immunostained with an antibody against the polyQ tract, from a small cohort of HD patients (see supplementary Table 2). Our analysis did not detect any occurrence of nucleolar polyQ aggregates in those cases, however we noted perinucleolar polyQ foci in neurons carrying nucleolar aggresome-like *cavities* in the frontal and temporal cortices from one HD case (supplementary Fig. 6).

### Nucleolar pTau aggresomes impact DNA but not rRNA levels

To investigate whether the presence and protein composition of nucleolar aggresomes influence nucleolar functional activity, we next measured rRNA immunofluorescence and DAPI fluorescence intensity in temporal cortex of aged control cases with central, large nucleolar aggresomes immunoreactive only for α-synuclein (among proteins examined here) versus AD cases with nucleolar aggresomes immunoreactive only for pTau (among proteins examined here).

**Fig. 9:**
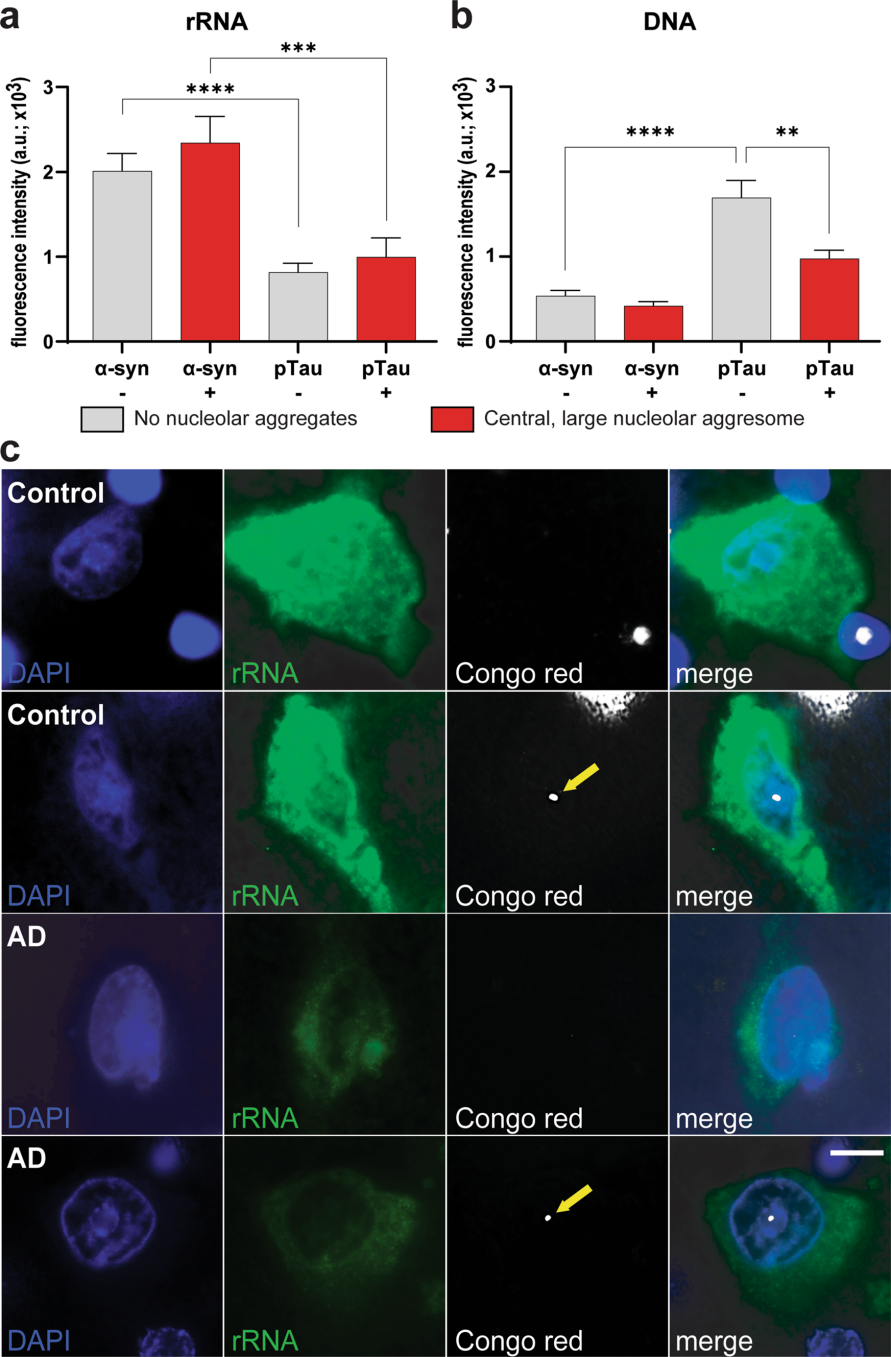
rRNA and DNA levels in neurons with and without nucleolar aggresomes in aged control and AD. Quantification of rRNA immunofluorescence (**a**) and DNA fluorescence (**b**) intensities in neurons with or without α-synuclein aggresome (from aged controls) and with or without pTau aggresomes (from AD cases), comparing those without any nucleolar aggregates (gray bars) to those containing a central, large (Congo red-positive) nucleolar aggresome (red bars). Neurons from aged control subjects with nucleolar α-synuclein aggresomes (Congo red-positive, red bar) or without nucleolar aggresomes (Congo red-negative, gray bar) exhibited significantly higher rRNA levels (as measured by the corrected rRNA immunofluorescence intensity) than AD neurons both in the absence (***p < 0.001) and presence (****p < 0.0001) of central, large pTau aggresomes (**a**). Neurons from aged controls with or without α-synuclein nucleolar aggresomes do not have different levels of DNA (as measured by DAPI corrected fluorescence), whereas AD neurons with pTau nucleolar aggresomes (Congo red-positive, red bar) have significantly lower DNA levels compared to AD neurons without any nucleolar aggregates (Congo red- negative, gray bar) (**p < 0.01) and aged controls neurons without any nucleolar aggregates (Congo red-negative, gray bar) (****p < 0.0001) (**b**). Representative images show DAPI (blue), neuronal rRNA (green) and Congo red-positive nucleolar aggresomes (yellow arrows) in neurons without (first and third row) and with (second and forth row) Congo red-positive nucleolar aggresomes. Congo red birefringence was imaged under brightfield with polarizing filters. Images were obtained from the temporal cortex of an aged control case (top rows) and an AD case (lower rows) using with ×100 objective. *a.u.*, arbitrary unit. Scale bar represents 10 µm (**c**)

Quantitative analysis of the rRNA immunofluorescence images in neurons with nucleolar aggresomes verified by Congo red staining versus neurons with no nucleolar amyloid aggregates revealed no significant difference in rRNA between neurons with or without either α-synuclein or pTau nucleolar aggresomes (Fig. 9a, c). As previously published [22, 30, 74], aged controls have higher rRNA levels compared with AD cases (Fig. 9a) with transcriptionally active neurons forming protein aggregates within nucleoli [48] functionally separated from the rRNA-transcribing compartments [50].

DNA damage is known to increase with age and contributes to neuronal loss in AD and synucleinopathies due to significant genomic variation [87]. To quantify DNA levels, DAPI fluorescence images were assessed in the same neurons revealing that nucleolar aggresome-free neurons in AD have increased DNA levels, as expected [6], which is mitigated in neurons with pTau nucleolar aggresomes (Fig. 9b, c). In contrast, there is no difference in DNA levels in controls with α-synuclein-positive nucleolar aggresomes (Fig. 9b) suggesting an impact on different nuclear functions.

## Discussion

The nucleolus consists of three well-defined, phase-separate subcompartments—GC, DFC, and FC—involved in ribosome biogenesis and protein trafficking, production and quality control for genome stability and cell responses [28, 33, 46, 92]. The nucleolus is known to be a highly complex fluid with changeable viscoelastic properties exhibiting features of both solids and liquids [33]. Our novel data in human neurons has identified a fourth type of compartment that is relatively frequent in adult human neurons, nucleolar *cavities* (Fig. 1). Surprisingly, these *cavities* often contain solid amyloids (Fig. 2). To the best of our knowledge, such nucleolar *cavities* containing amyloid aggregates have never been investigated previously in postmortem human brain and our data suggest these amyloid aggregates can fuse to form larger aggresomes (Fig. 1a) as previously shown in model systems [96]. The frequency of these larger aggresomes declines with neurodegeneration and neuronal loss, indicating that the loss of nucleolar aggresomes in neurons is detrimental.

Across most cohorts examined (aged controls, AD ± LATE, AD + LBD, LBD, FTLD-Tau, FTLD-TDP, and FTLD-FUS), multiple smaller nucleolar foci of amyloid nature were more abundant than nucleolar aggresomes (Fig. 3b). The predominance of these foci suggests that the physiological functions previously attributed to such reversible nucleolar aggregates, i.e., the transient sequestration of proteins in a liquid-state that permits later recovery [9, 28], are likely to be more frequently involved in these groups. Nucleolar aggresomes, although less frequent, were nonetheless detected in a subset of neurons across all cohorts (Fig. 3b). Prior work has shown that these inclusions are enriched for proteasome substrates and polyadenylated RNA and that their formation depends on nucleolar integrity [50]. Based on these findings, nucleolar aggresomes have been proposed to fulfill distinct roles, such as buffering nuclear proteotoxic stress by sequestering aggregation-prone proteins and RNAs into a defined compartment [50] and coupling protein quality control with nucleolar function [48]. Thus, although multiple smaller foci are the predominant form of nucleolar aggregation, the presence of nucleolar aggresomes in a subset of neurons indicates that distinct modes of nucleolar involvement in protein aggregation can occur in parallel within the same tissue.

Our follow-up experiments assessing known amyloid-prone proteins in neuropathological inclusion pathologies (Aβ, tau, α-synuclein, TDP-43, FUS, prion and dipeptide repeat proteins) found that only a subset were commonly found in nucleolar aggregates. α-Synuclein accumulation occurred frequently in these structures in the elderly with age-associated but not disease-progressing inclusion pathologies, whereas pTau accumulation in nucleolar aggresomes occurred with greater disease-associated accumulation of diverse inclusion pathologies. There was a correlation between the loss of α-synuclein and the accumulation of pTau in nucleolar aggresomes, suggesting that these two proteins independently deposit in nucleolar *cavities* under different circumstances along a normal aging to disease continuum. In contrast, pTDP-43-positive aggresomes characterized those with LATE but not other syndromes with TDP-43 inclusion pathologies, and the other aggregation-prone proteins were either not found or occurred in relatively few aggresomes in the cohorts of patients evaluated. These data suggest that only some aggregation-prone proteins are commonly found within adult neuronal nucleolar *cavities* and that they rarely occur together.

While the neuronal nucleolar location of such solid amyloid condensates has not been previously identified in adult human neurons, the formation of such amyloids is known and considered potentially functional rather than always pathogenic [14, 66]. Functional amyloids show a greater range of stabilities and lifetimes (evolved to be reversible when no longer required) compared to insoluble pathogenic fibrils, and perform five broad biological roles—structural amyloids maintain shape and support; reservoir amyloids store, protect, and moderate protein activity; information carrier amyloids maintain a particular state over time; function-suppressing amyloids reduce the activity of the soluble proteins from which they assemble; and signaling amyloids activate a process [83]. Dynamic, adaptive changes to nucleolar structure resembling neuronal nucleolar aggregates have been identified in response to stressful stimuli, with reversibly assembled proteins and RNAs accumulating in aggresomes and A bodies upon heat shock, acidosis or proteotoxic insults [31]. In terms of the frequency of the type of neuronal nucleolar amyloid aggregates, we have observed in adult neurons, multiple smaller nucleolar foci were the most prominent (Fig. 3b) and may represent foci of functional amyloids. For the amyloids that were the less frequently found in aggresomes, FUS has been previously identified as a functional amyloid with an absence of protofibril bundling to facilitate assembly and disassembly and abundant polar residues to maintain solvated monomers [83]. It is a multifunctional RNA-binding protein that dynamically localizes to the nucleolus in response to cellular stress [64, 106] and has been shown to regulate the expression of snoRNAs and changes in post-transcriptional modifications of rRNAs [32]. We show that FUS (and the two other FET proteins known to aggregate in inclusion pathologies, TAF15 and EWS) [59] only rarely form nucleolar amyloid aggregates. Aβ was also found in only a small proportion of nucleolar aggregates in adult human neurons, although it is sequestered into the nucleolus in cell models where it forms stable and immobile nucleolar aggresomes [48, 96]. Our data suggest that in the human brain, the sequestration of Aβ into the nucleolus is mainly found in transient multiple small foci in plaque-positive cases (Fig. 7a) and did not occur prior to the presence of amyloid plaques. This negates the hypothesis that the nucleolar sequestration of Aβ is a prelude to seeding extracellular plaque [96]. In fact, apart from pTau, we found no evidence that the deposition of nucleolar amyloids preceded diagnostic neuropathologies in the neurodegenerative diseases evaluated.

Until recently, it was controversial that α-synuclein had a nuclear localization, but studies have convincingly shown native and phosphorylated α-synuclein in the nucleus of neurons [43, 100] as well as in the nucleolus [7]. α-Synuclein binds and bends DNA to regulate DNA metabolism, transcription and repair [21, 84], chaperones histones to assemble and disassemble nucleosomes (essential for DNA replication, repair and transcription)[39], binds to highly stable RNA G-quadruplexes to accelerate its phase separation [65], and interacts with nucleolin (NCL) [38]—an abundant, multifunctional nucleolar protein [90]. The binding of α-synuclein to double stranded DNA stimulates its fibril formation [17, 34] with aggregated α-synuclein destabilizing DNA G-quadruplexes leading to unfolding [42]. In our study, the presence of nucleolar α-synuclein aggresomes in a high proportion of control subjects suggests a neuroprotective role (Fig. 6c). This was also consistent with the negative correlation between nucleolar α-synuclein aggregate formation stage and increasing neurofibrillary tangle burden across cohorts (Fig. 7e), supporting the concept that a loss of functional neuroprotective nucleolar α-synuclein in adult neurons leads to neurodegeneration [81, 84]. In fact, the nucleolar location of α-synuclein has been shown to protect melanoma cells from DNA damage [7] and autophagic cell death [95], and regulates DNA double strand break repairs in both transformed cells and primary cortical neurons [81]. These studies support the concept that nucleolar α-synuclein functionally contributes to genome stability, repair and transcription in neurons, and that a loss of such function is detrimental to neurons.

In contrast, tau is associated with nuclear integrity, altering chromatin architecture and nucleolar function [24, 26, 61, 78]. Tau undergoes phase separation with DNA, mononucleosomes and reconstituted nucleosome arrays to promote chromatin compaction, prevent DNA damage and digestion, and repress rRNA transcription [2, 24, 61, 102]. Tau also binds to RNA, RNA-binding proteins (RBP), ribosomal proteins, and transcription factors [24, 40, 55, 61]. Tau phosphorylation disrupts these associations [2, 24, 61] and, depending on the degree and sites of phosphorylation, forms functional amyloid fibrils (as seen with AT8 pTau antibody used in current analysis) or more highly phosphorylated pathogenic fibrils (seen with PHF pTau antibody) [62]. Tau phosphorylation leads to aberrant associations with nuclear RBPs and transcription factors to form amyloids and initiate nuclear dysfunction and gene instability [24, 68, 75]. Antibodies against total tau and non-phosphorylated tau have previously identified nucleolar sequestration of tau in cell models and in the human brain [61], and cell models have shown that pTau can also translocate to the nucleus [54, 80] and aggregate into A bodies under stress conditions [16]. There have been no previous studies describing nucleolar pTau aggresomes in human brain tissue and our study shows that such aggresomes (Fig. 4d-f) do not occur in asymptomatic cases (even those with age-related neurofibrillary tangle formation) but accumulate in all neurodegenerative disease cohorts with a higher stage of pTau aggregate formation associated with an increase in neuropathology (as seen for Aβ pathological load, Fig. 7c). We also show that in cases with AD that there is neuronal DNA instability and that the presence of nucleolar pTau aggresomes reduces such DNA instability, supporting a neuroprotective role for these structures. The reduced frequency of such aggresomes across many neurodegenerative diseases may contribute to the observed disruption to terminal neuronal gene differentiation that drives cell cycle re-entry and induces transposable element transcription and immune responses with consequent neuronal death [23, 25, 44, 98]. Together these data suggest that the accumulation of pTau in nucleolar aggresomes may contribute to neuronal survival through genomic stabilization in neurodegenerative diseases.

TDP-43 is a nuclear DNA and RNA-binding protein, and dense, spheroidal TDP-43 has been observed in neuronal nucleoli of sporadic ALS patients [82] indicative of nucleolar stress associated with diagnostic TDP-43 neuropathology [4, 5, 67]. TDP-43 is known to form a variety of functional high-density phase-separated assembles within the nucleus that are associated with RNA metabolism [47] although the nucleolar sequestration of phosphorylated TDP-43 has remained unexplored in human brain tissue. Our data show that pTDP-43 is sequestrated into central, large nucleolar aggresomes most prominent in cases with LATE or AD pathology (Fig. 7f), and absent in FTLD-TDP cases, even though the latter is considered a primary TDP-43 proteinopathy. This discrepancy suggests that the nucleolar sequestration of pTDP-43 is not predictive of diagnostic TDP-43 neuropathology itself, with the notable difference between cohorts being age. The aged human brain has reduced amounts of RBPs, especially spliceosome components, with more TDP-43 found in the cytoplasm due to stress granule recruitment [77]. As recently indicated, the nucleolus is a site of local protein translation when required [92] with amyloid formation as a potential protein store [83]. In the aged brain, pTDP-43 aggresomes could initially be a site of TDP-43 protein production and/or storage to compensate for this age-associated reduction. We also observed an increase in nucleolar pTDP-43 aggregation in these cohorts with increasing Aβ and tau pathology (Fig. 7g), consistent with a potentially compensatory mechanism prior to the development of considerable pTau aggresome formation and neurodegeneration. LATE has recently been distinguished by aberrant RNA splicing with cryptic exon inclusion [18], a phenomenon also observed in AD without LATE but with nuclear TDP-43 depletion [89]. Using Ribo-seq to identify mRNAs being transcribed into protein in cell models and CSF samples, some mis-spliced transcripts are known to be transcribed into de novo proteins in TDP-43 affected neurons [86]. The location of pTDP-43 aggresomes in the nucleolus where ribosomal biogenesis occurs is also consistent with such de novo protein translation. Whether such aberrant proteins also impact on neurodegeneration remains to be determined.

The other aggregation proteins assessed (prion protein, polyQ, and DRP) did not accumulate in neuronal nucleoli, but have been found in the nucleus of neurons. However, it is important to note that we did not use a prion antibody specific to the misfolded, disease-associated prion form (PrP^Sc^), which may exhibit different localization patterns. Also, polyQ and DRPs are de novo nuclear proteins only produced in those with enlarged repeat expansions. These proteins form intranuclear inclusions associated with gene loci not within nucleoli, polyQ depositing at the *HTT* gene locus [58, 73] and DRPs colocalizing with silent DNA loci [85]. Of note, neurons with intranuclear inclusions also exhibited nucleolar *cavities*, suggesting other amyloids than those assessed in the present study occur in these disorders. For more detailed discussions on the nuclear locations of these proteins, please see the supplementary discussion.

Our study provides compelling evidence that nucleolar sequestration and aggregation of amyloidogenic proteins is a widespread process in the human brain. We demonstrate that neurons with large nucleolar amyloidogenic aggresomes selectively degenerate as there is a negative association with neurodegeneration, and that such nucleolar aggresomes can contain α-synuclein, pTau or pTDP-43 and less often Aβ or FUS, but not prion or repeat expansion proteins. Apart from pTau, there was no correlation between the presence of proteins within the nucleolar aggresomes and their diagnostic neuropathological inclusions, refuting the concept that such nucleolar protein aggregates are seeds for their diagnostic cellular pathologies. α-Synuclein and pTau nucleolar aggresomes appear to occur on a continuum with nucleolar α-synuclein aggresomes found in neurons in most controls, suggesting a neuroprotective role, and decreasing with increasing pTau neuropathologies, while pTau aggresomes occurred in neurons across all neurodegenerative cohorts with greater pTau aggresome formation associated with increasing amounts of neurodegenerative pathologies. The assessment of neurons containing nucleolar pTau aggresomes versus those without revealed that such aggresomes were protective against genomic instability, at least in AD. Nucleolar aggresomes containing pTDP-43 distinguished cases with LATE or AD and were absent in all types of FTLD cases. The older age at onset of LATE compared to FTLD and the known age-related changes in cellular TDP-43 [77] are likely to contribute to nucleolar pTDP-43 aggregates potentially as compensation for reduced nuclear TDP-43 with age. This may contribute to LATE pathology. Our novel data provide compelling evidence that most human neurons contain nucleolar *cavities* with amyloidogenic proteins, and that such amyloids do not always appear to be detrimental and are likely to be functional in certain circumstances. The role of these structures and their relationship to nucleolar function and dysfunction require further research.

## Supplementary Information

Below is the link to the electronic supplementary material.

Supplementary file1 (PDF 4667 KB)

## Data Availability

Raw data generated in this study are available on Zenodo at 10.5281/zenodo.17547759.
