# Supplementary material for: Nucleolar aggregation of key neuropathological proteins in the postmortem neurodegenerative brain
Source: Acta Neuropathol. 2025 Dec 3;150(1):60. doi: 10.1007/s00401-025-02968-2 (PMC12675744; doi:10.1007/s00401-025-02968-2)
Supplement: Supplementary file 1 — Supplementary file1 (PDF 4667 KB) [file 401_2025_2968_MOESM1_ESM.pdf]

Supplementary materials for **'Nucleolar aggregation of key neuropathological proteins in the postmortem neurodegenerative brain'** by Guinevere F. Lourenco[1,2], Maria Elizabeth Torres-Pacheco[1,2], Yuhong Fu[1,2], Hongyun Li[1,2], Heather McCann[3], Claire E. Shepherd[3,4], Jillian J. Kril[1], Glenda M. Halliday

[1]Neuroscience, School of Medical Sciences, Faculty of Medicine and Health, University of Sydney, Sydney, Australia

[2]Brain and Mind Centre, University of Sydney, Sydney, Australia

[3]Neuroscience Research Australia, Sydney, Australia

[4]UNSW Medicine and Health School of Biomedical Sciences, UNSW Sydney, Sydney, Australia.

Supplementary table 1. Demographics and neuropathological information on the cohorts used for the immunohistochemistry analyses of nucleolar sequestration of neuropathological proteins. The analysis of amyloid nucleolar aggregates (figure 3) was performed on a subset of 5 cases from each cohort.

Pathological cohort	Subtype	Age (y)	Sex	PMD (h)	Duration (y)	Broe atrophy score	ABC score	Braak LB stage	Late TDP-43 stage
**N=7**	**-**	**80±4**	**7M:8F**	**19±4**	**-**	**-**	**14% no pathology**	**100% no pathology**	**100% no pathology**
Aged Control	-	64	F	5	-	-	A1B0C0	0	0
Aged Control	-	68	M	11	-	-	A0B0C0	0	0
Aged Control	-	84	M	22	-	-	A2B1C1	0	0
Aged Control	-	84	M	36	-	-	A1B0C0	0	0
Aged Control	-	84	F	16	-	-	A2B1C3	0	0
Aged Control	-	85	F	23	-	-	A2B0C1	0	0
Aged Control	-	93	F	21	-	-	A1B0C0	0	0
**N=8**	**-**	**89±2**	**7M:8F**	**26.5±4**	**-**	**-**	**0% no pathology**	**100% no pathology**	**0% no pathology**
LATE	-	79	M	8	-	-	A0B1C0	0	2
LATE	-	80	F	29	-	-	A1B1C0	0	2
LATE	-	88	F	31	-	-	A2B1C3	0	2
LATE	-	89	F	23	-	-	A1B1C0	0	2
LATE	-	89	M	24	-	-	A3B0C0	0	2
LATE	-	92	F	31	-	-	A0B1C0	0	2
LATE	-	93	M	46	-	-	A2B0C1	0	2
LATE	-	103	M	20	-	-	A3B1C1	0	2
**N=35**	**-**	**77±2**	**26M:9F**	**19±2**	**8±1**	**-**	**0% no pathology**	**100% no pathology**	**11% no pathology**
AD	-	64	F	18	5	-	A3B3C3	0	0
AD	-	67	M	6	2	-	A3B3C3	0	0
AD	-	70	M	23	7	-	A3B3C3	0	0
AD	-	80	F	32	10	-	A3B3C3	0	0
AD	LATE	55	M	20	2	-	A3B3C3	0	2
AD	LATE	56	M	19	4	-	A3B3C3	0	2
AD	LATE	61	M	19	5	-	A3B3C3	0	2
AD	LATE	62	M	24	6	-	A3B3C3	0	2
AD	LATE	66	M	9	9	-	A3B3C3	0	2
AD	LATE	66	M	20	23	-	A3B3C2	0	2
AD	LATE	68	M	6	10	-	A3B3C2	0	2
AD	LATE	69	M	18	5	-	A3B3C3	0	2
AD	LATE	69	M	19	9	-	A3B3C3	0	2
AD	LATE	70	M	8	13	-	A3B3C2	0	2
AD	LATE	70	M	35	4	-	A3B3C2	0	2
AD	LATE	71	M	35	12	-	A3B3C3	0	2
AD	LATE	73	M	26	7	-	A3B3C3	0	2
AD	LATE	74	M	35	4	-	A3B3C3	0	2
AD	LATE	75	F	14	8	-	A3B3C3	0	2

AD	LATE	79	M	67	7	-	A3B3C3	0	2
AD	LATE	80	M	19	7	-	A3B3C2	0	2
AD	LATE	80	M	12	9	-	A3B3C2	0	2
AD	LATE	83	M	14	11	-	A3B3C2	0	2
AD	LATE	84	M	10	7	-	A3B3C2	0	2
AD	LATE	85	F	10	5	-	A3B3C3	0	2
AD	LATE	86	F	15	12	-	A3B3C3	0	2
AD	LATE	86	M	9	11	-	A3B3C3	0	2
AD	LATE	87	M	40	15	-	A3B3C2	0	2
AD	LATE	88	F	19	8	-	A3B3C3	0	2
AD	LATE	90	M	7	4	-	A3B3C2	0	2
AD	LATE	91	F	6	7	-	A3B3C2	0	2
AD	LATE	92	M	9	10	-	A3B3C2	0	2
AD	LATE	98	F	11	6	-	A3B3C2	0	2
AD	LATE	99	M	17	3	-	A3B3C3	0	2
AD	LATE	100	F	3	11	-	A3B3C2	0	2
**N=9**	**4(6);4(5);1(4)**	**81±4**	**6M:3F**	**21±4**	**5±2**	**-**	**0% no pathology**	**0% no pathology**	**11% no pathology**
AD(+LBD)	cortical	66	M	10	14	-	A2B2C0	6	0
AD(+LBD)	cortical	73	F	7	5	-	A2B2C1	6	2
AD(+LBD)	cortical	75	M	27	3	-	A3B2C3	6	2
AD(+LBD)	cortical	87	F	41	1	-	A3B2C3	6	2
AD(+LBD)	limbic	70	M	8	1	-	A3B2C1	5	2
AD(+LBD)	limbic	79	M	9	11	-	A2B2C0	5	2
AD(+LBD)	limbic	84	M	12	2	-	A3B2C2	5	2
AD(+LBD)	limbic	90	M	5	7	-	A3B2C1	5	2
AD(+LBD)	brainstem	102	F	71	5	-	A3B2C2	4	2
**N=29**	**16(6);10(5);3(4)**	**78±1**	**24M:5F**	**21±3**	**13±1**	**-**	**7% no pathology**	**0% no pathology**	**31% no pathology**
LBD	cortical	67	M	36	11	-	A0B0C0	6	2
LBD	cortical	69	M	5	2	-	A3B1C2	6	2
LBD	cortical	69	M	28	3	-	A3B1C2	6	2
LBD	cortical	72	M	72	31	-	A1B1C0	6	0
LBD	cortical	74	M	16	8	-	A2B0C1	6	2
LBD	cortical	76	M	20	13	-	A0B1C0	6	0
LBD	cortical	77	M	61	30	-	A2B1C1	6	2
LBD	cortical	78	M	3	7	-	A1B2C0	6	2
LBD	cortical	78	F	17	10	-	A3B1C1	6	0
LBD	cortical	80	M	25	18	-	A0B0C0	6	0
LBD	cortical	81	M	13	15	-	A0B2C0	6	0
LBD	cortical	81	F	29	22	-	A0B1C0	6	2
LBD	cortical	82	M	22	7	-	A2B1C1	6	2
LBD	cortical	86	M	16	28	-	A3B1C3	6	2
LBD	cortical	90	M	14	10	-	A1B0C0	6	2

LBD	cortical	92	M	46	8	-	A3B1C3	6	2
LBD	limbic	67	M	21	19	-	A2B0C1	5	0
LBD	limbic	71	M	25	15	-	A0B1C0	5	2
LBD	limbic	71	M	24	7	-	A1B1C1	5	0
LBD	limbic	77	M	18	11	-	A1B1C1	5	0
LBD	limbic	78	M	5	10	-	A0B2C0	5	2
LBD	limbic	81	M	5	9	-	A3B1C2	5	2
LBD	limbic	82	F	9	8	-	A0B2C0	5	2
LBD	limbic	83	M	7	11	-	A2B0C1	5	2
LBD	limbic	83	F	18	20	-	A2B1C1	5	2
LBD	limbic	85	M	34	15	-	A1B2C0	5	2
LBD	brainstem	72	M	5	10	-	A1B2C1	4	0
LBD	brainstem	72	M	8	9	-	A0B2C0	4	2
LBD	brainstem	80	F	6	7	-	A1B2C0	4	2
**N=20**	**5PiD;5GGT; 5CBD:5PSP**	**74±2**	**11M:9F**	**27±3**	**7±1**	**1.5±0.3**	**5% no pathology**	**100% no pathology**	**25% no pathology**
FTLD - Tau	Pick's disease	66	F	22	6	3	A0B1C0	0	0
FTLD - Tau	Pick's disease	67	F	31	8	2	A2B1C2	0	0
FTLD - Tau	Pick's disease	72	M	31	13	4	A3B0C1	0	2
FTLD - Tau	Pick's disease	73	M	11	7	4	A0B0C0	0	2
FTLD - Tau	Pick's disease	82	M	43	5	2	A0B2C0	0	2
FTLD - Tau	GGT	71	M	19	3	1	A1B2C0	0	2
FTLD - Tau	GGT	76	F	10	5	1	A0B0C0	0	0
FTLD - Tau	GGT	82	F	41	9	0	A2B0C0	0	2
FTLD - Tau	GGT	89	F	11	3	1	A1B0C1	0	2
FTLD - Tau	GGT	90	F	30	13	1	A2B2C2	0	2
FTLD - Tau	CBD	56	M	23	7	2	A0B3C0	0	2
FTLD - Tau	CBD	66	F	10	4	1	A1B0C0	0	0
FTLD - Tau	CBD	69	M	24	7	2	A0B1C0	0	2
FTLD - Tau	CBD	79	M	23	4	2	A0B1C0	0	2
FTLD - Tau	CBD	87	F	36	6	1	A1B2C0	0	2
FTLD - Tau	PSP	69	F	19	7	1	A1B1C1	0	2
FTLD - Tau	PSP	70	M	18	11	0	A0B1C0	0	0
FTLD - Tau	PSP	70	M	41	4	0	A1B1C0	0	2
FTLD - Tau	PSP	71	M	58	7	1	A1B2C0	0	2
FTLD - Tau	PSP	76	M	30	2	0	A2B1C0	0	2
**N=15**	**8(A/B);7C**	**74±2**	**11M:4F**	**23±4**	**8±1**	**2.2±0.2**	**53% no pathology**	**100% no pathology**	**0% no pathology**
FTLD - TDP	Type A/B	58	F	18	3	2	A0B0C0	0	3
FTLD - TDP	Type A/B	60	M	28	2	2	A0B0C0	0	3
FTLD - TDP	Type A/B	71	M	44	4	1	A0B1C0	0	3
FTLD - TDP	Type A/B	74	F	8	2	1	A0B0C0	0	3
FTLD - TDP	Type A/B	74	M	20	7	2	A0B0C0	0	3
FTLD - TDP	Type A/B	76	M	9	4	3	A3B1C3	0	3

FTLD - TDP	Type A/B	84	F	17	8	3	A3B0C3	0	3
FTLD - TDP	Type A/B	86	M	10	7	1	A1B2C1	0	3
FTLD - TDP	Type C	68	M	44	12	3	A0B0C0	0	3
FTLD - TDP	Type C	68	M	6	8	3	A0B0C0	0	3
FTLD - TDP	Type C	69	M	18	11	2	A1B1C0	0	3
FTLD - TDP	Type C	72	M	5.5	13	3	A0B0C0	0	3
FTLD - TDP	Type C	76	M	24	9	2	A0B0C0	0	3
FTLD - TDP	Type C	82	M	66	17	3	A2B2C0	0	3
FTLD - TDP	Type C	86	F	25	7	2	A2B0C1	0	3
**N=5**	**4aFTLDU; 1NIFID**	**57±5**	**4M:1F**	**11±3**	**5±1**	**3±0.3**	**80% no pathology**	**100% no pathology**	**100% no pathology**
FTLD-FUS	aFTLDu	68	F	22	7	4	A0B0C0	0	0
FTLD-FUS	aFTLDu	62	M	8	2	3	A0B0C0	0	0
FTLD-FUS	aFTLDu	39	M	5	6	3	A0B0C0	0	0
FTLD-FUS	aFTLDu	50	M	5	4	3	A0B0C0	0	0
FTLD-FUS	NIFID	64	M	15	6	2	A1B2C0	0	0

AD, Alzheimer's disease; *aFTLDu*, atypical FTLD with ubiquitinated inclusions; *CBD*, corticobasal degeneration; *DLB*, dementia with Lewy bodies; *FTLD*, frontotemporal lobar degeneration; *GGT*, globular glial tauopathy; *LBD*, Lewy body disease; *MND*, motor neurone disease; *NIFID*, neuronal intermediate filament inclusion disease; *PD*, Parkinson's disease; *PSP*, progressive supranuclear palsy.

Supplementary table 2. Demographics and neuropathological information on the genetic cohorts.

Pathological cohort	Subtype / Stage	Age (y)	Sex	PMD (h)	Duration (y)	Broe atrophy score	ABC score	Braak LB stage	Late TDP-43 stage
**N=17**	**-**	**68±8.6**	**8M:9F**	**28±25.5**	**6±3.6**	**1±1**	**24% no pathology**	**88% no pathology**	**0% no pathology**
c9orf72 FTLD-TDP	type A	65	F	24	5	0	A0B0C0	0	3
c9orf72 FTLD-TDP	type A	65	F	5	3	3	A0B2C0	0	3
c9orf72 FTLD-TDP	type B	49	F	26	2	0	A0B0C0	0	3
c9orf72 FTLD-TDP	type B	55	M	118	3	1	A0B0C0	0	3
c9orf72 FTLD-TDP	type B	61	M	11	5	2	A1B0C0	0	3
c9orf72 FTLD-TDP	type B	66	M	9	6	2	A2B1C0	0	3
c9orf72 FTLD-TDP	type B	81	F	14	8	3	A1B2C0	0	3
c9orf72 FTLD-TDP	type B	68	F	17	16	2	A2B0C0	0	3
c9orf72 FTLD-TDP	type A	75	F	26	12	1	A2B2C3	0	3
c9orf72 FTLD-TDP	type A	61	M	39	7	2	A0B2C0	0	3
c9orf72 FTLD-TDP	type B	69	F	24	8	1	A0B1C0	0	3
c9orf72 FTLD-TDP	type A	69	M	37	5	1	A0B0C0	0	3
c9orf72 FTLD-TDP	type A	67	F	22	2	2	A0B2C0	0	3
c9orf72 FTLD-TDP	type A	77	F	22	6	0	A0B2C0	6	3
c9orf72 FTLD-TDP	type A	83	M	13	5	2	A0B2C0	0	3
c9orf72 FTLD-TDP	type A	67	M	25	4	N/A	A0B1C0	0	3
c9orf72 FTLD-TDP	type A	71	M	44	4	N/A	A0B1C0	Amygdala predominant	3
**N=11**	**-**	**59±9.2**	**5M:6F**	**24±8.8**	**2±0.9**	**-**	**45% no pathology**	**91% no pathology**	**0% no pathology**
c9orf72 ALS	4	55	F	23	2	N/A	A2B1C1	0	3
c9orf72 ALS	4	56	M	22	3	N/A	A0B0C0	0	3
c9orf72 ALS	4	74	M	22	1	N/A	A0B0C0	0	3
c9orf72 ALS	4	49	M	42	2	N/A	A0B0C0	0	3
c9orf72 ALS	4	59	F	20	2	N/A	A0B0C0	0	3
c9orf72 ALS	4	67	M	26	2	N/A	A2B0C0	0	3
c9orf72 ALS	3	65	M	23	1	N/A	A1B1C0	0	2
c9orf72 ALS	4	45	F	31	1	N/A	A0B0C0	0	3
c9orf72 ALS	4	72	F	14	2	N/A	A0B2C0	1	3
c9orf72 ALS	4	54	F	9	4	N/A	A1B0C0	0	3
c9orf72 ALS	4	57	F	31	2	N/A	A0B1C0	0	3
**N=5**	**-**	**60±11**	**3M:2F**	**25±7**	**13±3**	**-**	**40% no pathology**	**100% no pathology**	**100% no pathology**
HD	4*	58	F	26	20	N/A	A0B1C0	0	0
HD	2*	65	M	6	4	N/A	A0B0C0	0	0
HD	3*	20	F	16	13	N/A	A0B0C0	0	0
HD	2*	91	M	27	15	N/A	A2B0C1	0	0
HD	1*	64	M	48	13	N/A	A1B2C0	0	0

ALS, Amyotrophic lateral sclerosis; *HD*, Huntington's disease; FTLD, frontotemporal lobar degeneration, * Vonsattel stage.

Supplementary table 3. Primary antibodies used for immunohistochemistry.

Antibody to	Species	Isotype	Manufacturer	Catalogue No.	Dilution
Nucleolar and nuclear proteins					
RPA194 (POLR1A)	Mouse	Monoclonal IgG_{2a}	Santa Cruz Biotechnology	sc-48385	1:25
Fibrillarin (38F3)	Mouse	Monoclonal IgG_1	GeneTex	GTX24566	1:35
NPM1	Rabbit	Polyclonal	Merk	HPA011384	1:800
CUG-BP1 (EPR8298(B))	Rabbit	Monoclonal IgG	Abcam	ab129115	1:400
rRNA (Y10b)	Mouse	Monoclonal IgG_{3k}	Thermo Fisher Scientific	MA1-16628	1:20
Pathological proteins					
Aβ (6F/3D)	Mouse	Monoclonal $IgG_{1\kappa}$	Dako	M0872	1:200
Phospho-tau (AT8; S202/T205)	Mouse	Monoclonal $IgG_{1\kappa}$	Thermo Fisher Scientific	MN1020	1:1,500
α-synuclein (clone 42)	Mouse	Monoclonal IgG_1	BD Transduction Laboratories	610787	1:7,000
Phospho-α-synuclein (S129; EP1536Y))	Rabbit	Monoclonal IgG	Abcam	ab51253	1:1,000
Phospho-TDP-43 (S409/410; clone 11-9)	Mouse	Monoclonal IgG_1	Cosmo Bio USA	TIP-PTD-M01	1:80,000
C9ORF72/C9RANT (poly-GA; clone 5E9)	Mouse	Monoclonal $IgG_{1\kappa}$	Sigma-Aldrich	MAB889	1:1,000
FUS	Rabbit	Polyclonal	Sigma-Aldrich	HPA008784	1:200
	Mouse	Monoclonal IgG_1l	Proteintech	60160-1-Ig	1:1,000
TAF15 (TAFII68)	Rabbit	Polyclonal	Bethyl Laboratories	IHC-00094	1:200
EWS (G-5)	Mouse	Monoclonal $IgG_{1\kappa}$	Santa Cruz Biotechnology	Sc-28327	1:400
Polyglutamine expansion (5TF1-1C2)	Mouse	Monoclonal $IgG_{1\kappa}$	Merk	MAB1574	1:1,000
Prion protein (clone 3F4)	Mouse	Monoclonal IgG_{2a}	Merk	MAB1562	1:100

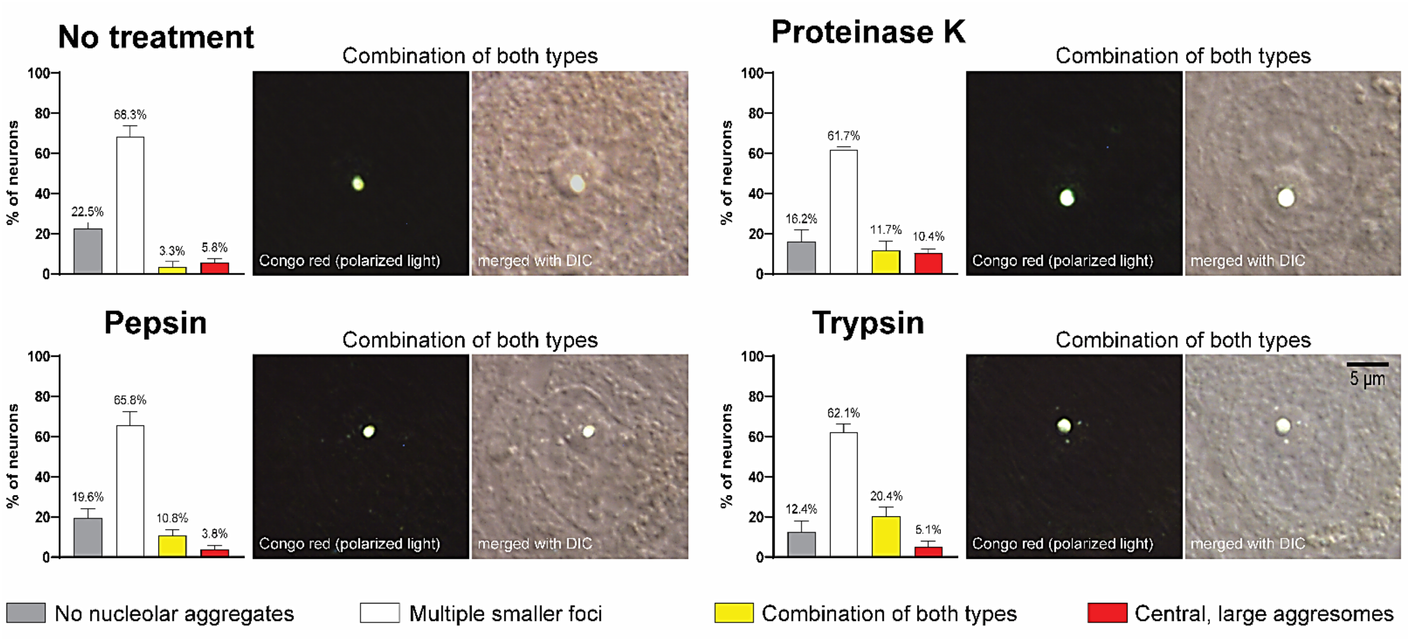

Supplementary figure 1. Amyloid nucleolar aggregates are resistant to degradation by proteolytic enzymes. Temporal cortex from one AD LATE case and one case with mixed pathology, and superior frontal cortex from two other cases with mixed pathology were used to determine whether nucleolar aggregates are resistant to proteolytic treatment (mean percentage and standard errors for nucleolar aggregate free and aggregate type determined). FFPE tissue sections from the four cases were submitted to digestion with proteinase K [20 ug/ml], pepsin (ab64201), and trypsin (ab259256), followed by Congo red staining. These were compared to sections that received only Congo red staining (no treatment). There was no significant difference in the frequency of neurons without nucleolar aggregates between the untreated samples and those treated with any proteolytic enzyme. Similarly, no statistically significant changes were observed in the frequency of neurons with multiple smaller nucleolar foci, central, large aggregates, or with a combination of both types between the untreated and protease-treated samples. It was noted that the protease-treated groups exhibited a slightly lower mean percentage of neurons without nucleolar inclusions and with multiple smaller foci. This is likely due to the more aggressive effects of enzymatic digestion on neurons without nucleolar aggregates or very small foci, resulting in these cells being less frequently counted in the treated samples. In contrast, the protease-treated samples exhibited a slightly higher mean percentage of neurons carrying a combination of both types of nucleolar aggregates. This suggests that, as neurons without aggregates and neurons with multiple smaller foci are more susceptible to proteolytic treatment and thus underrepresented in the final counts, neurons with a combination of smaller and larger aggregates are relatively overrepresented in the treated samples. Representative images were obtained from the superior frontal cortex of a case with mixed pathology using brightfield microscopy with polarizer filter (left side panels) and DIC (right side panels) with 100x magnification.

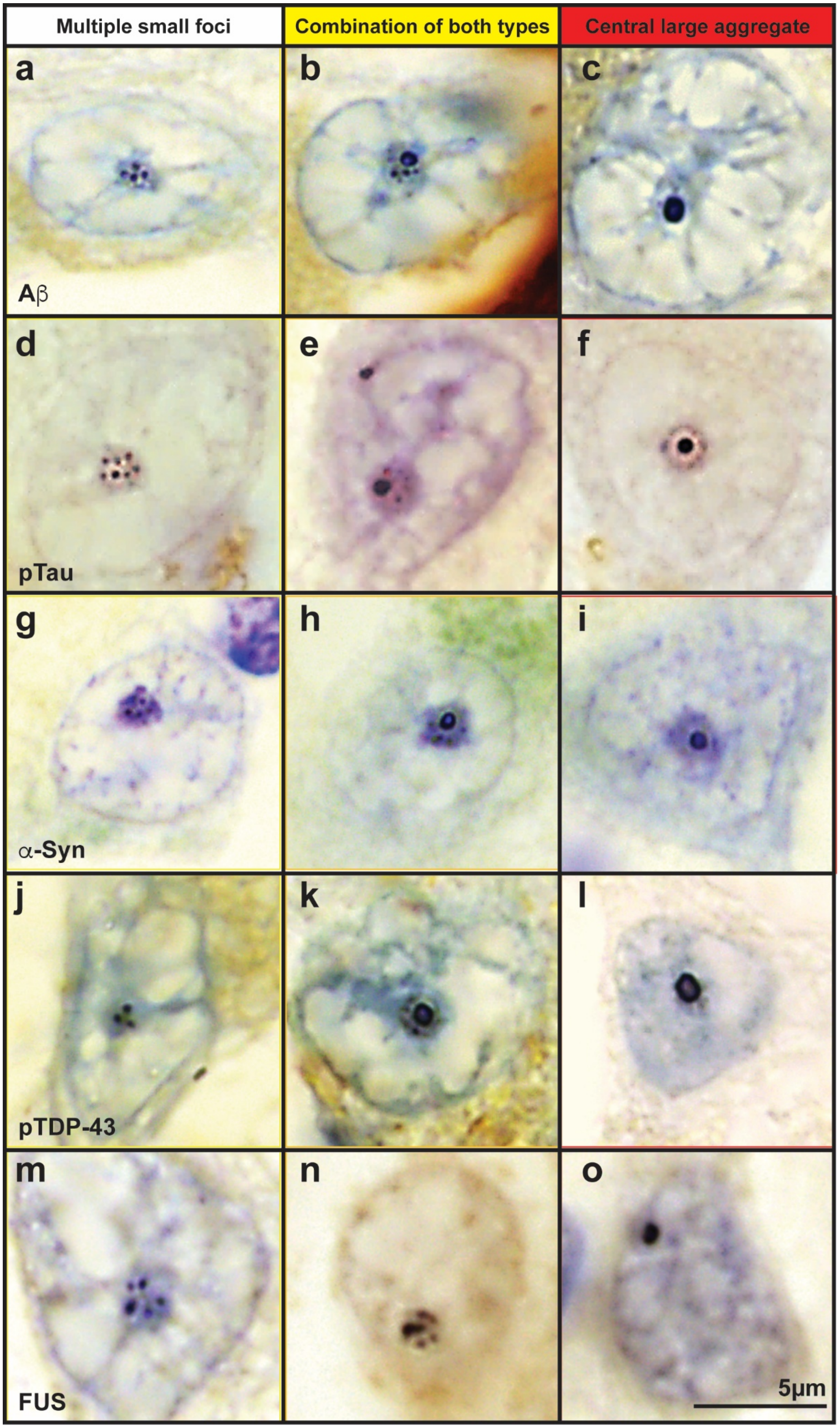

Multiple small foci
Combination of both types
Central large aggregate
a
b
c
Aβ
d
e
f
pTau
g
h
i
α-Syn
j
k
l
pTDP-43
m
n
o
FUS
5μm

Supplementary figure 2. Nucleolar sequestration of key neuropathological proteins in the neurodegenerative brain. FFPE tissue sections from postmortem brain from patients with neurodegenerative diseases were immunostained with antibodies against five neuropathological proteins – Aβ (**a**, **b**, **c**), phosphorylated tau (pTau **d**, **e**, **f**), α-synuclein (**g**, **h**, **i**), phosphorylated TDP-43 (pTDP-43 **j**, **k**, **l**), and FUS (**m**, **n**, **o**). All neuropathological proteins examined are sequestered into the three identified types of nucleolar aggregates: *multiple smaller foci* (**a**, **d**, **g**, **j**, **m**), *a combination of both types* (**b**, **e**, **h**, **k**, **n**), and *a central large aggregate* (**c**, **f**, **i**, **l**, **o**). Representative images for Aβ were obtained from the frontal cortex of AD cases; images from the anterior cingulate cortex from AD cases were used for both pTau and α-synuclein; for pTDP-43 images were obtained from the superior frontal cortex of FTLD-TDP cases; and for FUS images were obtained from the hippocampus, striatum, and motor cortex of FTLD-FUS cases. All panels are original images from neuronal nuclei where the nucleolus can be visually identified. These images were acquired using brightfield microscopy with 100x objective.

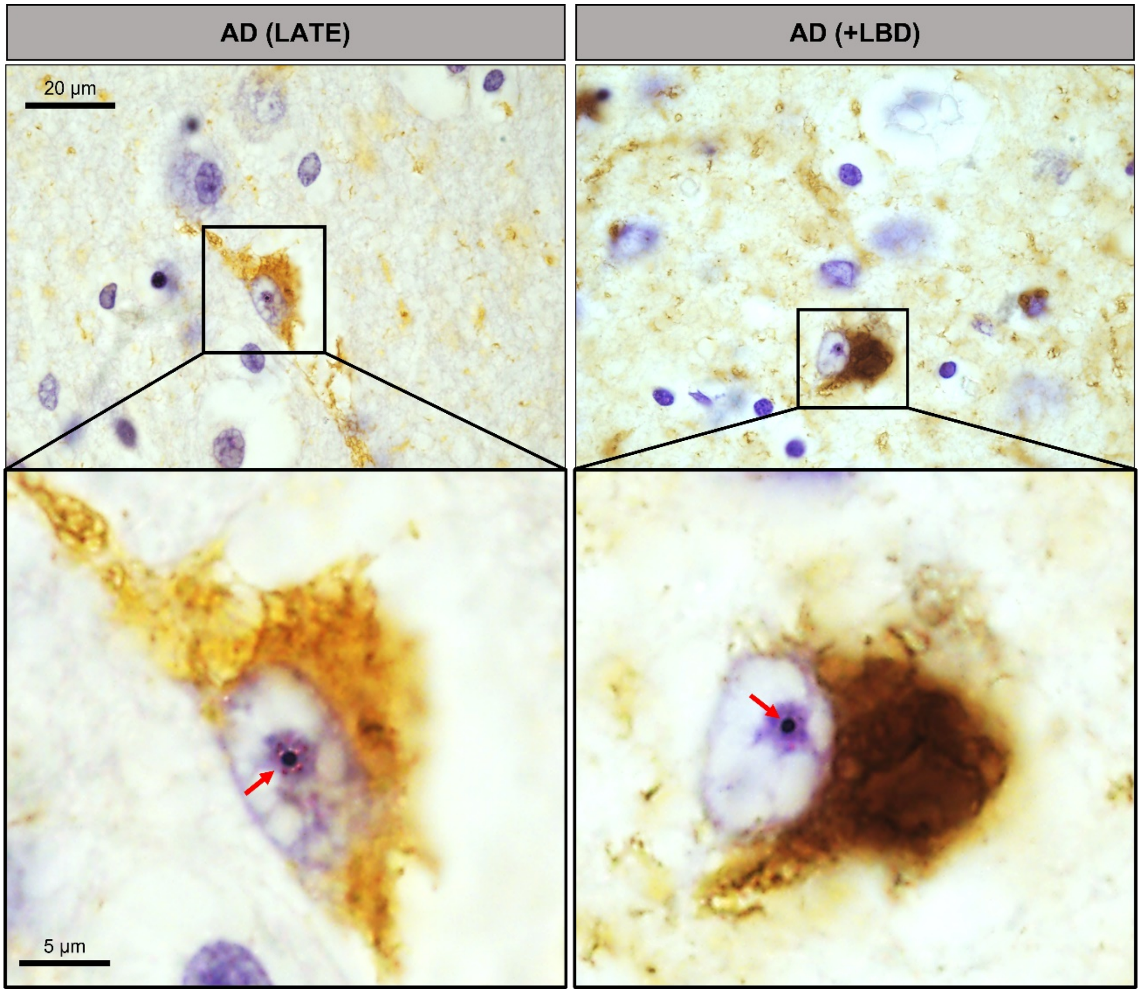

Supplementary figure 3. Central large aggregate of phosphorylated tau protein in AD (LATE) and AD (+LBD) brains. Central large nucleolar aggregates of pTau resembling solid-like aggresomes (red arrows) were also found in neuronal cells carrying cytoplasmic pTau. Representative images were obtained from the anterior cingulate cortex of a AD (LATE) case (left panels), and from the temporal cortex of an AD (+LBD) case (right panels). Images were acquired using brightfield microscopy with 100x objective.

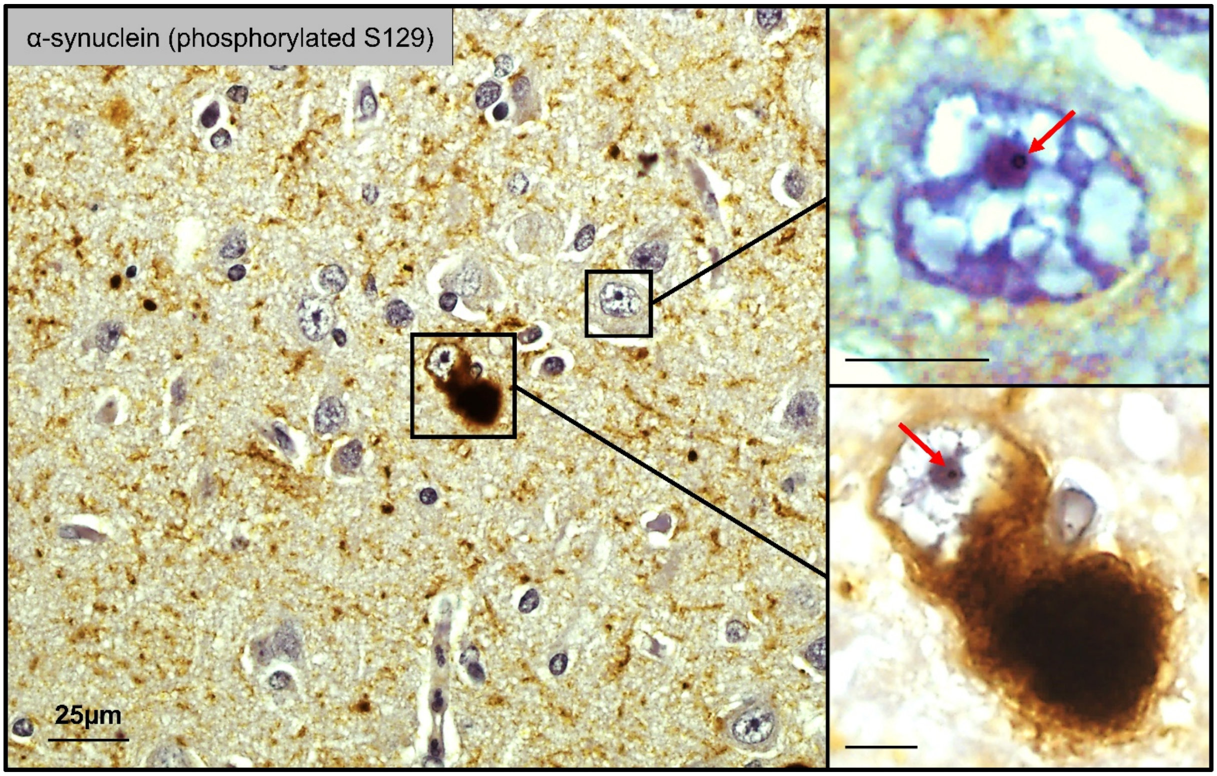

Supplementary figure 4. Nucleolar sequestration of phosphorylated α-synuclein in LBD brain. FFPE tissue sections immunostained with an antibody against α-synuclein phosphorylated on Ser129 [EP1536Y] from the anterior cingulate or temporal cortex of a small cohort of LBD cases (N = 5) were available. Nucleolar sequestration of phosphorylated α-synuclein was found in three cases (red arrows), regardless of the presence of intraneuronal aggregates of α-synuclein (lower inset). Representative images were obtained from the anterior cingulate cortex from a PD case, and were acquired using brightfield microscope with 40x (overview panel) and 100x (insets) objectives. Scale bars for insets represent 5μm.

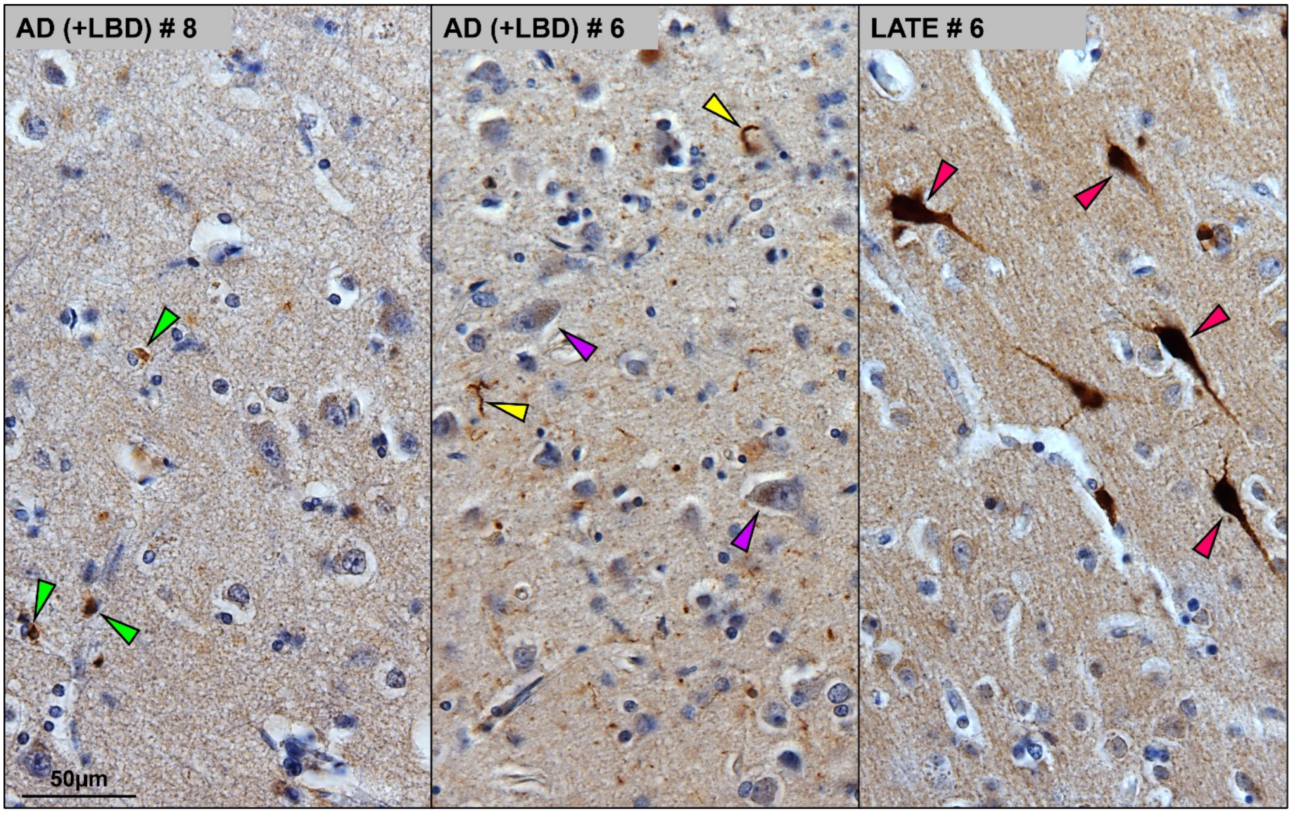

Supplementary figure 5. PrP^C immunostaining does not detect nucleolar aggregates in FFPE brain tissue. Immunohistochemistry using antibody against PrP^C revealed varied staining patterns, including cases with no staining, and cases with discrete glial inclusions (green arrowheads), PrP^C-positive neuritic processes (yellow arrowheads), faint neuronal cytoplasmic staining (purple arrowheads), and/or strong neuronal staining (pink arrowheads). No nucleolar aggregates of PrP^C were observed in any brain region from any cases from all cohorts examined. Representative images show PrP^C immunostaining in the frontal cortex of an AD (+LBD) case (left panel), in the anterior cingulate cortex of another AD (+LBD) case (middle panel), and in the temporal cortex of a LATE case (right panel).

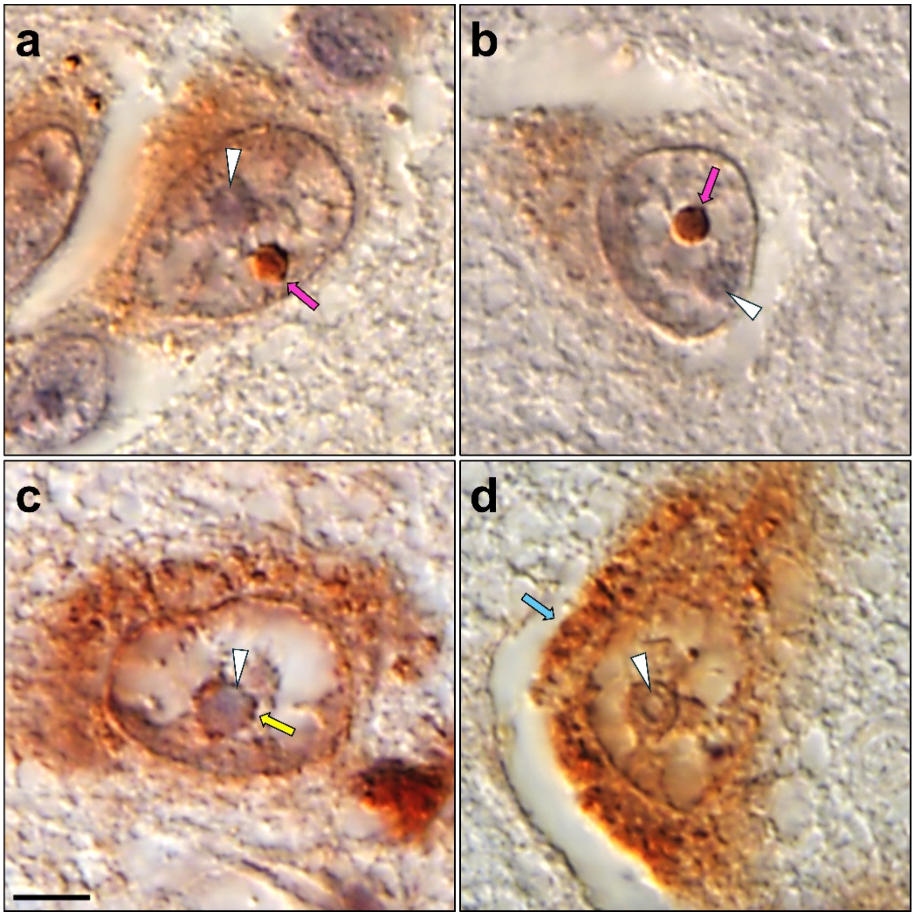

Supplementary figure 6. PolyQ inclusion bodies in HD neurons containing nucleolar *cavities*. Neurons from the frontal and temporal cortices of a HD patient carrying nucleolar aggresome-like *cavities* (white arrowheads) display polyQ tract-positive intranuclear inclusions (**a** and **b**, pink arrows). Perinucleolar polyQ foci (**c**, yellow arrow) and cytoplasmic deposition (**d**, blue arrow) in HD neurons carrying nucleolar *cavities* (white arrowheads) were also observed. Representative images were obtained from the temporal cortex (**a** and **b**) and frontal (**c** and **d**) of one HD patient and acquired using DIC microscopy with 100x objective lens. Scale bar represents 5µm.

Supplementary discussion on proteins that did not accumulate in neuronal nucleoli.

Prion protein

PrP^C is not a classic nucleic acid-binding protein with mutations or membrane associations required for fibril formation [4]. However, it does contain cryptic nuclear localization signals, which may facilitate its translocation to the nucleus under certain conditions [7]. Not surprisingly, PrP^C has been reported to be localized to the nucleus in cell models [2, 14] but a nucleolar localization has never been demonstrated. In our study, we did not detect PrP^C within the nucleolus in any of the brain regions examined. It is important to note, however, that we did not include cases of prion disease and did not use an antibody specific to the misfolded, disease-associated prion form (PrP^{Sc}), which may exhibit different localization patterns. Therefore, while our data suggests an absence of nucleolar prion aggregates under the conditions studied, further investigation using prion disease brain tissue and antibodies against PrP^{Sc} would be necessary to fully assess the nucleolar involvement of prion proteins in disease cohorts.

Repeat expansion proteins

The other aggregation-prone proteins assessed were those for genetic expansions known to make *de novo* proteins that pathologically aggregate in the nucleus, that is polyQs from *HTT* expansions and DPRs from *c9orf72* expansions. Normal HTT has long been recognized as an essential protein for proper nucleolar function [8], but the expanded CAG repeat RNA from the abnormal *HTT* gene directly interacts with nucleolin interfering with its ability to bind to the rRNA promoter [16] and dispersing nucleophosmin 1 [13] disrupting nucleolar function. The expanded CAG repeat RNA undergoes phase transitions and translation, and along with polyQ, deposits in nuclear inclusions at the *HTT* gene loci and not in nucleoli [9, 10]. Our analysis confirms this, revealing the absence of nucleolar polyQ tract in three brain regions from five HD cases (supplementary figure 6). It is possible that the nucleolar stress induced by expanded CAG repeat RNAs [1, 13] prevents polyQ-HTT from accessing the nucleolus and forming aggregates. However, given the presence of nucleolar *cavities* within the nucleolus from polyQ-positive HD neurons (supplementary figure 6), it is more likely that the exclusion of polyQ tracts from the nucleolus results from the sequestration

and immobilization of other amyloidogenic proteins into nucleolar aggregates and aggresomes. Nuclear translational machinery [15] or protein quality control proteins [3] are recruited into the nucleolus under stress conditions and for the elimination of inclusions and may accumulate in such amyloid bodies in HD. DRPs are abnormally translated from intronic expansions in the *c9orf72* gene, and like the expanded *HTT* gene, both the expanded repeat RNAs and novel translated proteins accumulate in diagnostic nuclear inclusions outside of the nucleolus [5, 12]. Unlike HTT, C9orf72 is not a nuclear protein [11] and the nuclear location of the expanded hexanucleotide RNAs and the DRPs are not collocated, but the nuclear DRP aggregates do colocalize with silent DNA [12]. In line with current literature, our analysis confirms the absence of nucleolar poly(GA) in Purkinje cells from the cerebellum of *c9orf72*-related FTLD and ALS patients. We hypothesized and verified that the link between poly(GA) deposition and nucleolar dysfunction may potentially be the consistent co-occurrence of nucleolar *cavities* in neurons with poly(GA)$^+$ inclusions (figure 8). In the model proposed by Frottin and colleagues [6], DPR proteins lead to nucleolar dysfunction in cell lines by entering the nucleolus from the nucleoplasm and modulating its phase-transition properties. However, collectively, our observations suggest an alternative scenario for neurons with *c9orf72* expansions: the sequestration and immobilization of amyloidogenic proteins into nucleolar aggresomes potentially cause liquid-to-solid phase transition, preventing poly(GA) peptides from entering the nucleolus and, therefore, causing these DPR proteins to self-aggregate elsewhere in the neuron. This may help explain not only the consistent exclusion of poly(GA) from the nucleolus but also the nucleolar toxicity associated with the presence of poly(GA)$^+$ deposition. It will be important to determine which proteins are sequestered into nucleolar aggresomes in those neurons and unveil their contribution to the formation of DPR aggregates.

Supplementary references

1 Aviner R, Lee TT, Masto VB, Li KH, Andino R, Frydman J (2024) Polyglutamine-mediated ribotoxicity disrupts proteostasis and stress responses in Huntington's disease. Nat Cell Biol 26: 892-902 Doi 10.1038/s41556-024-01414-x

2 Banik P, Ray K, Kamps J, Chen QY, Luesch H, Winklhofer KF, Tatzelt J (2024) VCP/p97 mediates nuclear targeting of non-ER-imported prion protein to maintain proteostasis. Life Sci Alliance 7: e202302456 Doi 10.26508/lsa.202302456

3 Brunello L, Polanowska J, Le Tareau L, Maghames C, Georget V, Guette C, Chaoui K, Balor S, O'Donohue MF, Bousquet MPet al (2025) A nuclear protein quality control system for elimination of nucleolus-related inclusions. EMBO J 44: 801-823 Doi 10.1038/s44318-024-00333-9

4 do Amaral MJ, Freire MHO, Almeida MS, Pinheiro AS, Cordeiro Y (2023) Phase separation of the mammalian prion protein: Physiological and pathological perspectives. J Neurochem 166: 58-75 Doi 10.1111/jnc.15586

5 Frottin F, Perez-Berlanga M, Hartl FU, Hipp MS (2021) Multiple pathways of toxicity induced by C9orf72 dipeptide repeat aggregates and G(4)C(2) RNA in a cellular model. Elife 10: e62718 Doi 10.7554/eLife.62718

6 Frottin F, Schueder F, Tiwary S, Gupta R, Korner R, Schlichthaerle T, Cox J, Jungmann R, Hartl FU, Hipp MS (2019) The nucleolus functions as a phase-separated protein quality control compartment. Science 365: 342-347 Doi 10.1126/science.aaw9157

7 Gu Y, Hinnerwisch J, Fredricks R, Kalepu S, Mishra RS, Singh N (2003) Identification of cryptic nuclear localization signals in the prion protein. Neurobiol Dis 12: 133-149 Doi 10.1016/s0969-9961(02)00014-1

8 Hilditch-Maguire P, Trettel F, Passani LA, Auerbach A, Persichetti F, MacDonald ME (2000) Huntingtin: an iron-regulated protein essential for normal nuclear and perinuclear organelles. Hum Mol Genet 9: 2789-2797 Doi 10.1093/hmg/9.19.2789

9 Ly S, Didiot MC, Ferguson CM, Coles AH, Miller R, Chase K, Echeverria D, Wang F, Sadri-Vakili G, Aronin Net al (2022) Mutant huntingtin messenger RNA forms neuronal nuclear clusters in rodent and human brains. Brain Commun 4: fcac248 Doi 10.1093/braincomms/fcac248

10 Pan Y, Lu J, Feng X, Lu S, Yang Y, Yang G, Tan S, Wang L, Li P, Luo Set al (2023) Gelation of cytoplasmic expanded CAG RNA repeats suppresses global protein synthesis. Nat Chem Biol 19: 1372-1383 Doi 10.1038/s41589-023-01384-5

11 Pang W, Hu F (2021) Cellular and physiological functions of C9ORF72 and implications for ALS/FTD. J Neurochem 157: 334-350 Doi 10.1111/jnc.15255

12 Schludi MH, May S, Grasser FA, Rentzsch K, Kremmer E, Kupper C, Klopstock T, German Consortium for Frontotemporal Lobar D, Bavarian Brain Banking A, Arzberger Tet al (2015) Distribution of dipeptide repeat proteins in cellular models and C9orf72 mutation cases suggests link to transcriptional silencing. Acta Neuropathol 130: 537-555 Doi 10.1007/s00401-015-1450-z

13 Sonmez A, Mustafa R, Ryll ST, Tuorto F, Wacheul L, Ponti D, Litke C, Hering T, Kojer K, Koch Jet al (2021) Nucleolar stress controls mutant Huntington toxicity and monitors Huntington's disease progression. Cell Death Dis 12: 1139 Doi 10.1038/s41419-021-04432-x

14 Strom A, Wang GS, Picketts DJ, Reimer R, Stuke AW, Scott FW (2011) Cellular prion protein localizes to the nucleus of endocrine and neuronal cells and interacts with structural chromatin components. Eur J Cell Biol 90: 414-419 Doi 10.1016/j.ejcb.2010.11.015

15 Theodoridis PR, Bokros M, Marijan D, Balukoff NC, Wang D, Kirk CC, Budine TD, Goldsmith HD, Wang M, Audas TEet al (2021) Local translation in nuclear condensate amyloid bodies. Proc Natl Acad Sci U S A 118: e2014457118 Doi 10.1073/pnas.2014457118

16 Tsoi H, Lau TC, Tsang SY, Lau KF, Chan HY (2012) CAG expansion induces nucleolar stress in polyglutamine diseases. Proc Natl Acad Sci U S A 109: 13428-13433 Doi 10.1073/pnas.1204089109
